# Identification of transient receptor potential channel genes from the swimming crab, *Portunus Trituberculatus*, and their expression profiles under acute temperature stress

**DOI:** 10.1186/s12864-024-09973-x

**Published:** 2024-01-17

**Authors:** Yichen Qian, Qiaoling Yu, Jun Zhang, Yaoyao Han, Xi Xie, Dongfa Zhu

**Affiliations:** Key Laboratory of Aquacultural Biotechnology Ministry of Education, School of Marine Sciences, Ningbo University, Ningbo, China

**Keywords:** *Portunus Trituberculatus*, Transient receptor potential channels, Temperature stress, Molecular characterization, Expression analysis

## Abstract

**Background:**

Temperature is an important environment factor that is critical to the survival and growth of crustaceans. However, the mechanisms by which crustaceans detect changes in temperature are still unclear. The transient receptor potential (TRP) channels are non-selective cation channels well known for properties in temperature sensation. However, comprehensive understandings on TRP channels as well as their temperature sensing functions are still lacking in crustaceans.

**Results:**

In this study, a total of 26 *TRP* genes were identified in the swimming crab, *Portunus trituberculatus*, which can be classified into TRPA, TRPC, TRPP, TRPM, TRPML, TRPN and TRPV. Tissue expression analysis revealed a wide distribution of these *TRP* genes in *P. trituberculatus*, and antennules, neural tissues, and ovaries were the most commonly expressed tissues. To investigate the responsiveness of *TRP* genes to the temperature change, 18 *TRPs* were selected to detect their expression after high and low temperature stress. The results showed that 12 *TRPs* showed induced gene expression in both high and low temperature groups, while 3 were down-regulated in the low temperature group, and 3 showed no change in expression in either group.

**Conclusions:**

This study characterized the *TRP* family genes in *P. trituberculatus*, and explored their involvement in response to temperature stress. Our results will enhance overall understanding of crustacean TRP channels and their possible functions.

**Supplementary Information:**

The online version contains supplementary material available at 10.1186/s12864-024-09973-x.

## Background

Temperature is one of the main environmental variables that significantly affect the physiological characteristics of aquatic and terrestrial animals [1]. Crustaceans are poikilothermic animals, so water temperature is critical to their survival and growth [2]. Crabs reared at higher water temperatures often have a shorter intermolt period and a faster growth rate, and vice versa [3, 4]. Due to global warming and the frequent occurrence of climate extremes, shrimps and crabs are constantly exposed to temperature changes during propagating and cultivating processes [5]. Investigating the mechanisms underlying the signals of a temperature change as perceived by crustaceans could provide a theoretical guidance for their scientific farming. However, such mechanisms remain largely unknown.

Transient receptor potential (TRP) channels are ideal candidates when it comes to research in this area, as they are widely known for sensing changes in temperature, osmolarity, and mechanical stimuli in mammals and insects [6]. TRP channels share a common structure with six transmembrane helical segments, two variable and intracellular amino (-NH2) and a carboxy (-COOH) terminal cytosolic domain, and form a pore region between TM5 and TM6, which gives most TRP channels the non-selective cation property that have high Ca^2+^ permeability [7]. Based on the homology of amino acid sequences, TRP channels in animals can be classified into nine subfamilies that belong to two groups [8]. Group 1 includes subfamilies TRPA (ankyrin), TRPC (canonical), TRPN (nompC, or no mechanoreceptor potential C), TRPM (melastatin), TRPS (soromelastatin), TRPV (vanilloid), and TRPVL (vanilloid-like), while Group 2 involves subfamilies TRPP (polycystin or polycystic kidney disease) and TRPML (mucolipin) [9]. The TRPA, TRPV, TRPVL, TRPC, and TRPN subfamilies are characterized by variable numbers of ankyrin repeats (ARs) at their N-terminus, which are essential for their interactions with ligands and protein partners [10]. Besides, TRPC, TRPN, and TRPM proteins are hallmarked with a WKxxR motif called TRP domain (or TRP box) in their C-terminals of the TM domain, which is vital for channel activation [11]. There are other structural domains that also affect the function of TRP channels, such as the coiled-coil domain, nudix hydrolase domain, and EF hand, but the types of structural domains vary between different TRP channels [6].

TRP channels can be gated by temperature and have extensive thermosensory functions in the animalia [12, 13]. In mammals, detection of temperature variations is proposed to be attributed by some channels in TRPV, TRPM, TRPC, and TRPA subfamily [13]. While in arthropods, members of TRPP and TRPN were also included [14]. The directionality of these TRP channels for sensing temperature has been particular well studied in the *Drosophila melanogaster*. Briefly, TRPC, TRPM, TRPP, and TRPN are involved in cold-sensing [14, 15], and TRPA is essential for heat avoidance [16, 17]. However, whether a TRP channel detects heat or cold is not conserved among species, even within the same class. For instance, the *Drosophila* TRPA1 was demonstrated as a heat sensor, but in rice planthoppers (*Sogatella furcifera*), this channel is involved in cold avoidance [18, 19]. It has been proposed that the inconsistency in function of TRP proteins in different species may be related to sequence discrepancy in their specific transmembrane domains [13, 20].

In recent years, the presence of TRP channels have been reported in several crustaceans. *Daphnia pulex* is a microcrustacean that has 14 TRP channels, with all the subfamilies represented [21, 22]. The barnacle, *Balanus improvisus*, has 13 TRP channels in the transcriptomes of its antennules [23]. Among the decapod, *Homarus americanus* has 7 TRP channels in the nervous system, while *Cancer borealis* has 6 TRP channels [22]. TRP channels have also been identified in the transcriptomes of antennular lateral flagella and dactyl of *Panulirus argus*, *Callinectes sapidus*, and *Procambarus clarkii* [24]. However, some homologs representing subfamilies of TRP channels are still missing currently in many crustaceans. More importantly, the temperature-sensing function of the *TRP* family genes in crustaceans have been barely reported, except for *TRPA1*, which exhibits possible temperature sensor properties in *P. clarkii* and *Eriocheir sinensis* [25, 26].

The swimming crab, *Portunus trituberculatus* is a commercially important crustacean species that have been artificially propagated and cultivated [27]. In this study, the *TRP* family genes of *P. trituberculatus* were identified, and their tissue distributions were analyzed by RT-PCR. To investigate the responsiveness of *TRP* genes to the temperature change, the high and low temperature stress experiments were performed. The findings of this study provide valuable information for future studies on the role of *TRP* genes in temperature sensing.

## Results

### Identification of TRP families

**Fig. 1:**
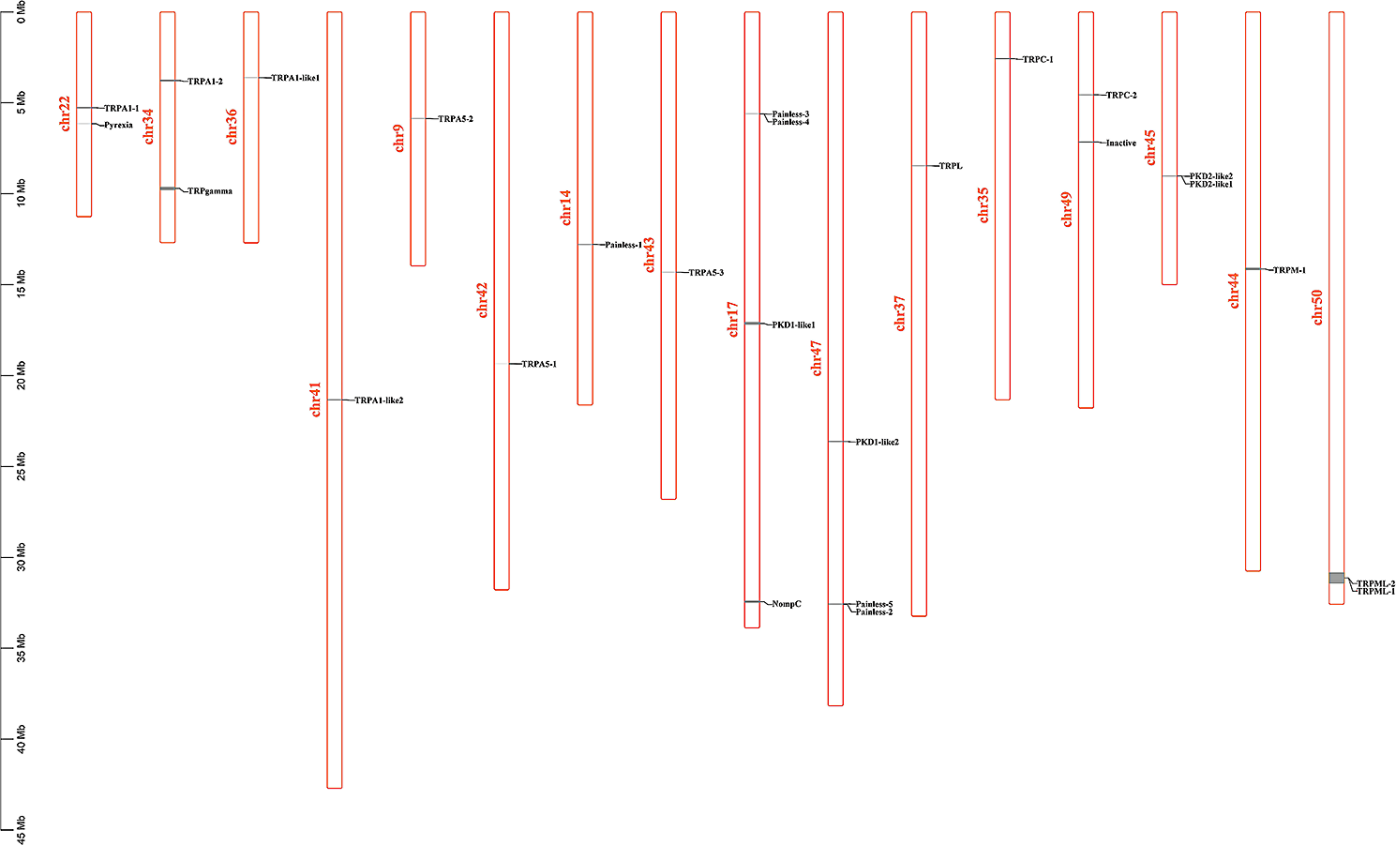
Distribution of TRP genes in chromosome of *P. trituberculatus*. Twenty-six TRP genes were mapped on the sixteen *P. trituberculatus* chromosomes. The scale on the left is in million bases (Mb). Chromosome numbers are indicated at the left of each vertical bar

**Fig. 2:**
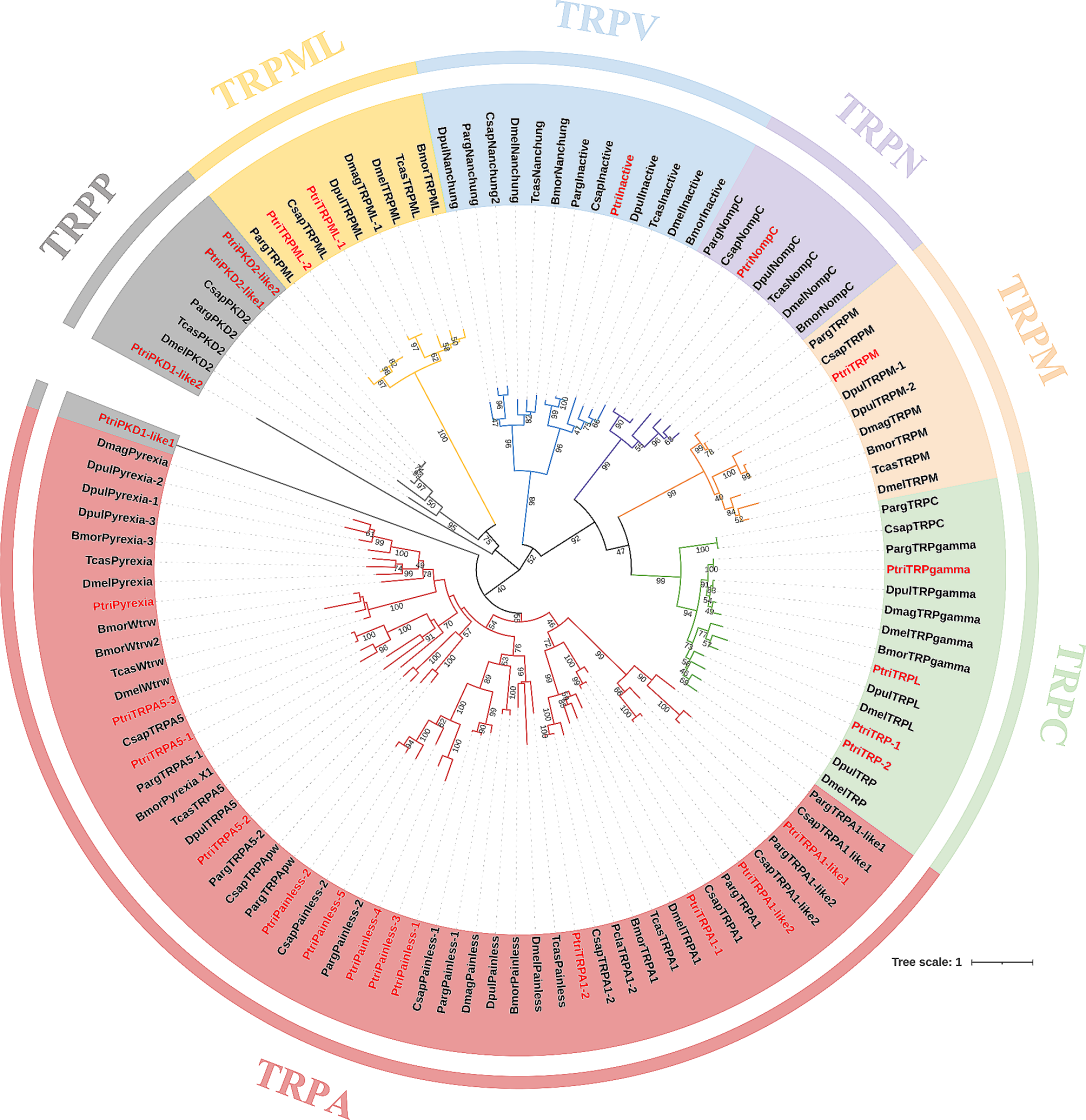
Maximum likelihood phylogenetic tree of TRP channels. Ptri, *Portunus trituberculatus*; Parg, *Panulirus argus*; Csap, *Callinectes sapidus*; Pcla, *Procambarus clarkii*; Dpul, *Daphnia pulex*; Dmag, *Daphnia magna*; Dmel, *Drosophila melanogaster*; Bmor, *Bombyx mori*; Tcas, *Tribolium castaneum*. The species sequence accession numbers are listed in Additional File 1. Various subfamilies of TRP channels are indicated by different colors: TRPA subfamily (red), TRPC (green), TRPN (purple), TRPM (pale orange), TRPML (yellow), TRPP (grey) and TRPV (blue). *P. trituberculatus* has several homologues to each subfamily of TRP channels

A total of 26 *TRP* sequences were identified from the *P. trituberculatus* using keywords screening of our transcriptomic databases, and were named according to the annotations of highest homology sequences. They can be divided into seven TRP subfamilies, which are TRPA, TRPC, TRPN, TRPM, TRPV, TRPML, and TRPP (Table 1). The *TRP* genes were predicted to locate nonuniformly on sixteen chromosomes (Fig. 1). Furthermore, to assign the candidates to TRP subfamilies, we constructed maximum-likelihood phylogeny which consisted of TRPs from insect and crustacean species (Fig. 2).

**Table 1:** Putative Transient receptor potential (TRP) channels in the transcriptomes of *P. trituberculatus*

TRP families	Members	Transcriptome size (bp)	ORF size (aa)	BLAST matches species	E-value	Identity	Accession number
TRPA	TRPA1-1	4500	1253	*Portunus trituberculatus*	0.00E + 00	91.34%	XP_045125037.1
	TRPA1-2	6716	1198	*Portunus trituberculatus*	0.00E + 00	99.92%	XP_045116162.1
	TRPA1-like1	3846	1062	*Portunus trituberculatus*	0.00E + 00	76.23%	XP_045126889.1
	TRPA1-like2	4461	1145	*Portunus trituberculatus*	0.00E + 00	90.48%	XP_045132316.1
	TRPA5-1	4664	853	*Portunus trituberculatus*	0.00E + 00	99.88%	XP_045133994.1
	TRPA5-2	2999	811	*Portunus trituberculatus*	0.00E + 00	99.75%	XP_045106459.1
	TRPA5-3	3032	861	*Portunus trituberculatus*	0.00E + 00	86.31%	XP_045135190.1
	Pyrexia	5225	991	*Portunus trituberculatus*	0.00E + 00	99.90%	MPC07503.1
	Painless-1	5435	926	*Portunus trituberculatus*	0.00E + 00	99.89%	XP_045109188.1
	Painless-2	4542	910	*Portunus trituberculatus*	0.00E + 00	99.78%	XP_045101153.1
	Painless-3	3086	857	*Portunus trituberculatus*	0.00E + 00	99.51%	MPC26245.1
	Painless-4	3578	945	*Portunus trituberculatus*	4.00E-177	48.39%	MPC26245.1
	Painless-5	4393	968	*Portunus trituberculatus*	0.00E + 00	70.01%	XP_045101150.1
TRPC	TRPgamma	5795	857	*Portunus trituberculatus*	0.00E + 00	98.04%	XP_045125085.1
	TRPL	5693	1361	*Portunus trituberculatus*	0.00E + 00	96.66%	XP_045127472.1
	TRP-1	4780	1165	*Portunus trituberculatus*	0.00E + 00	99.91%	XP_045126101.1
	TRP-2	3885	1091	*Portunus trituberculatus*	0.00E + 00	99.91%	XP_045103271.1
TRPP	PKD2-like1	3089	786	*Portunus trituberculatus*	0.00E + 00	100%	XP_045137074.1
	PKD2-like2	2839	792	*Portunus trituberculatus*	0.00E + 00	96.97%	XP_045137075.1
	PKD1-like1	9745	2021	*Portunus trituberculatus*	0.00E + 00	100%	XP_045111836.1
	PKD1-like2	14,560	3227	*Portunus trituberculatus*	0.00E + 00	100%	XP_045139437.1
TRPV	Inactive	5815	1173	*Portunus trituberculatus*	0.00E + 00	95.91%	XP_045103264.1
TRPM	TRPM	7045	1408	*Portunus trituberculatus*	0.00E + 00	99.93%	XP_045135393.1
TRPML	TRPML-1	2739	662	*Portunus trituberculatus*	0.00E + 00	100%	XP_045104760.1
	TRPML-2	2735	678	*Portunus trituberculatus*	0.00E + 00	92.48%	XP_045104760.1
TRPN	NompC	7969	1810	*Portunus trituberculatus*	0.00E + 00	98.62%	XP_045112035.1

**Fig. 3:**
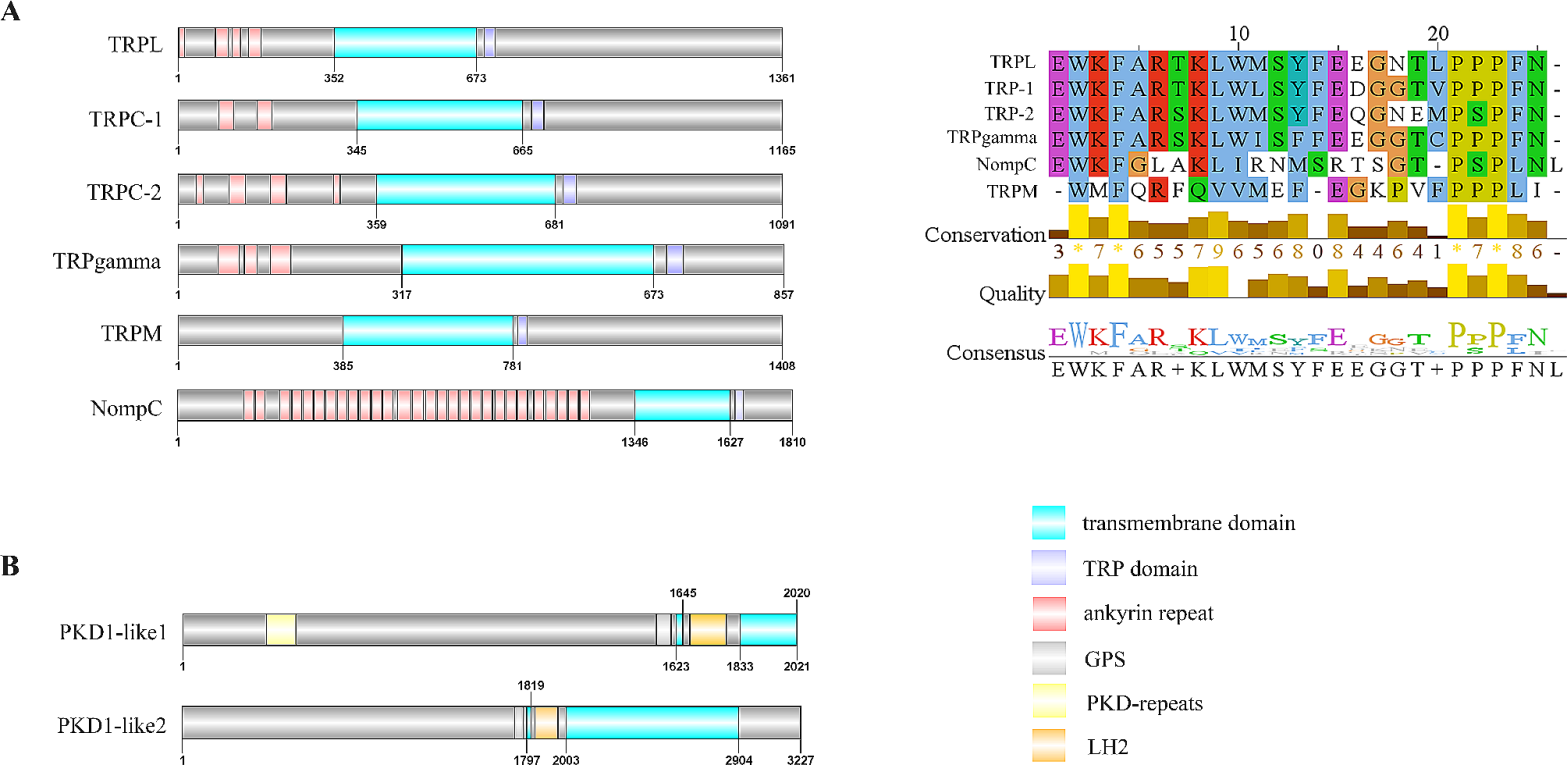
The domain organization of TRPC, TRPN, TRPM, and TRPP subfamilies. Schematic diagrams show structures of TRP channels, including transmembrane domains, ankyrin repeats, GPS, TRP domain, PKD-repeats, and LH2. Predicted TRP domain amino acid sequence of TRPL, TRPC-1, TRPC-2, TRPgamma, TRPM, and NompC have been aligned using Clustal X, shown conserved sequence motifs by Jalview 2.11.2

The TRPA subfamily of *P. trituberculatus* includes 13 members consist of two TRPA1, two TRPA1-like, five Painless, three TRPA5, and one Pyrexia. Of the two TRPA1 sequences, the PtriTRPA1-1 homologues seems to be only present in crustaceans, whereas the PtriTRPA1-2 homologues were found in both insects and crustaceans. Four transcripts were assigned to the classical TRPC subfamily, which are characterized by a conserved TRP domain, including PtriTRPL, PtriTRPgamma, PtriTRP-1 and PtriTRP-2. The TRPC identified in *Panulirus argus* and *Callinectes sapidus* seems absent in our data as no similar sequences were found. Only one sequence was identified in each of TRPN, TRPV, and TRPM subfamily, designated as PtriNompC, PtriInactive, and PtriTRPM respectively. Among them, the PtriNompC has 28 ankyrin repeats (ARs) in the N-terminal region and a TRP domain in the C-terminal region (Fig. 3A).

As for the TRPs from group 2, four sequences (PtriPKD1-like1, PtriPKD1-like2, PtriPKD2-like1 and PtriPKD2-like2) were assigned to TRPP subfamily and two (PtriTRPML-1 and PtriTRPML-2) to TRPML subfamily (Fig. 2). Normally, the PKD1-like proteins were defined by more than six transmembrane segments and several additional domains, such as GPS, PKD-repeats, and LH2 [8]. Both PtriPKD1-like sequences were characterized by these typical domains, and the PtriPKD1-like2 contains 12 transmembrane regions, while the sequence of PtriPKD1-like1 has an incomplete 3’-region, which only contains 5 transmembrane regions (Fig. 3B). The incomplete PtriPKD1-like1 was not clustered with TRPP homologues, but showed more closely related to the TRPA proteins (Fig. 2). The *PtriPKD2-like1* and *PtriPKD2-like2* were identical in most of their sequences, with a 24 bp deletion in *PtriPKD2-like1*, suggesting they might be produced by alternative splicing. *PtriTRPML-1* and *PtriTRPML-2* are two splice variants in which *PtriTRPML-1* is 48 bp deletion than *PtriTRPML-2*.

### Tissue distribution of ***TRP*** genes

**Fig. 4:**
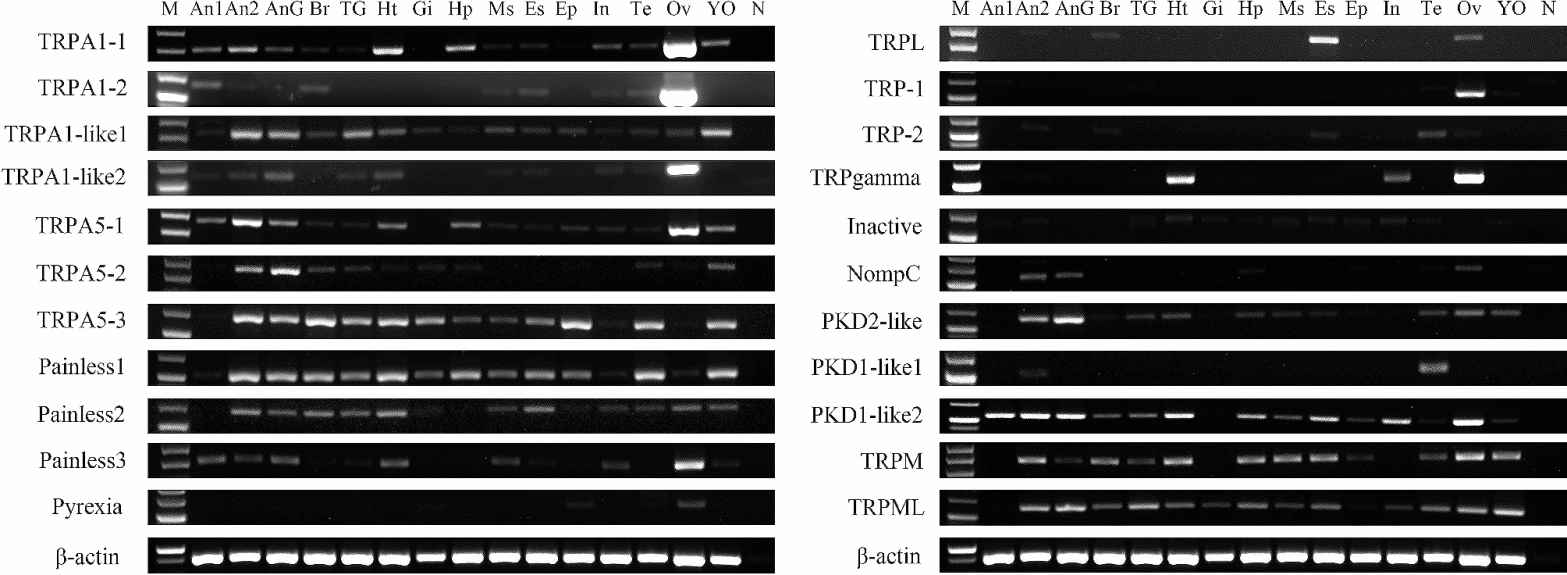
Tissue distribution of *P. trituberculatus* TRP transcripts. Twenty-two TRP transcripts were detected in the fifteen tissues from *P. trituberculatus*. M, DNA Marker; An1, Antenna 1; An2, Antenna 2; AnG, antennal gland; Br, brain; TG, thoracic ganglion; Ht, heart; Gi, gill; Hp, hepatopancreas; Ms, muscle; Es, eyestalk; Ep, epidermis; In, intestine; Ov, ovary; YO, Y-organ; Te, testis; N, negative control (representing no template in PCR). β-actin was used as the reference gene

The mRNA expression levels of predicted *TRP* genes varied in crab tissues. Considering that sequence differences between the splice variants cannot be accurately distinguished by PCR, the result of *TRPML* in Fig. 4 represents the common expression pattern of *PtriTRPML-1* and *PtriTRPML-2*, and *PtriPKD2-like* represents the common expression pattern of *PtriPKD2-like1* and *PtriPKD2-like2*.

Results from RT-PCR showed that 15 *TRP* transcripts were expressed in the sensory structures (Fig. 4). Among them, *PtriTRPA1-2* was biased expressed in the antennules. On the other hand, *PtriTRPA1-like1*, *PtriTRPA1-like2*, *PtriTRPA5-2*, *PtriTRPA5-3*, *PtriPainless-1*, *PtriPainless-2*, *PtriPainless-3*, *PtriNompC*, *PtriPKD2-like*, *PtriTRPM*, and *PtriTRPML* were biased presented in the antennae. *PtriTRPA1-1*, *PtriTRPA5-1*, *PtriPainless-3*, and *PtriPKD1-like2* were expressed in both the antennules and antennae. The expression of *PtriTRPM*, *PtriTRPML*, *PtriPKD1-like2*, *PtriTRPA5-3*, *PtriPainless-1*, and *PtriPainless-2* was relatively higher in neural tissues such as the thoracic ganglion, eyestalk, and brain. Besides, the results also showed that some of the *TRP* transcripts were present in the antennal glands. Notably, some *TRP* transcripts were expressed at extremely high levels in the ovary, and these include the TRPA1-1, TRPA1-2, and TRPA1-like2.

### Expression of ***TRP*** genes under acute temperature stress

**Fig. 5:**
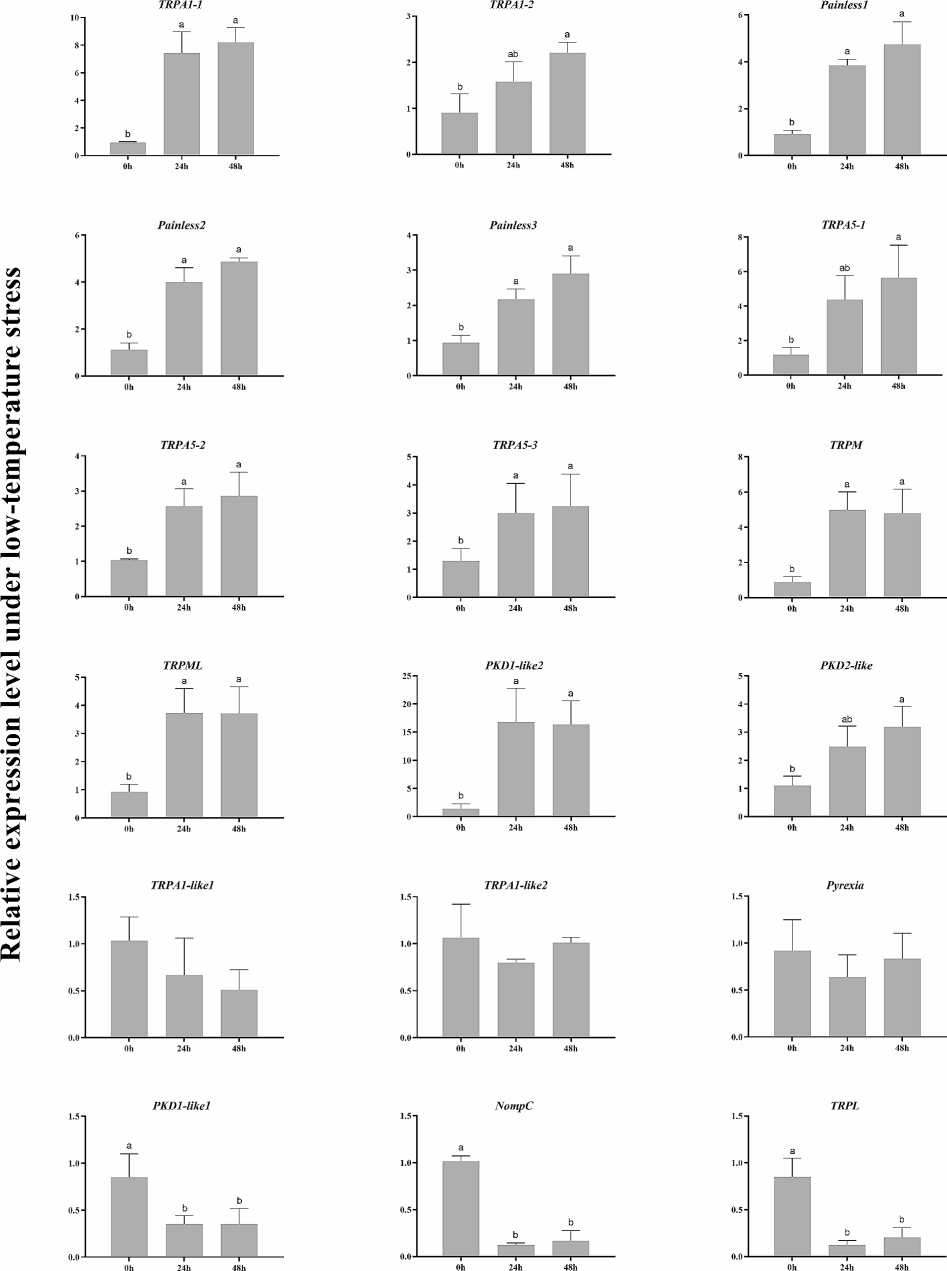
Relative expression of *TRP* genes under low-temperature stress in *P. trituberculatus*. Data are presented as mean ± SD (*n* = 3). Significant differences among groups are indicated by different letter labels (one-way ANOVA, followed by post hoc Tukey’s multiple-group comparison, *P* < 0.05)

**Fig. 6:**
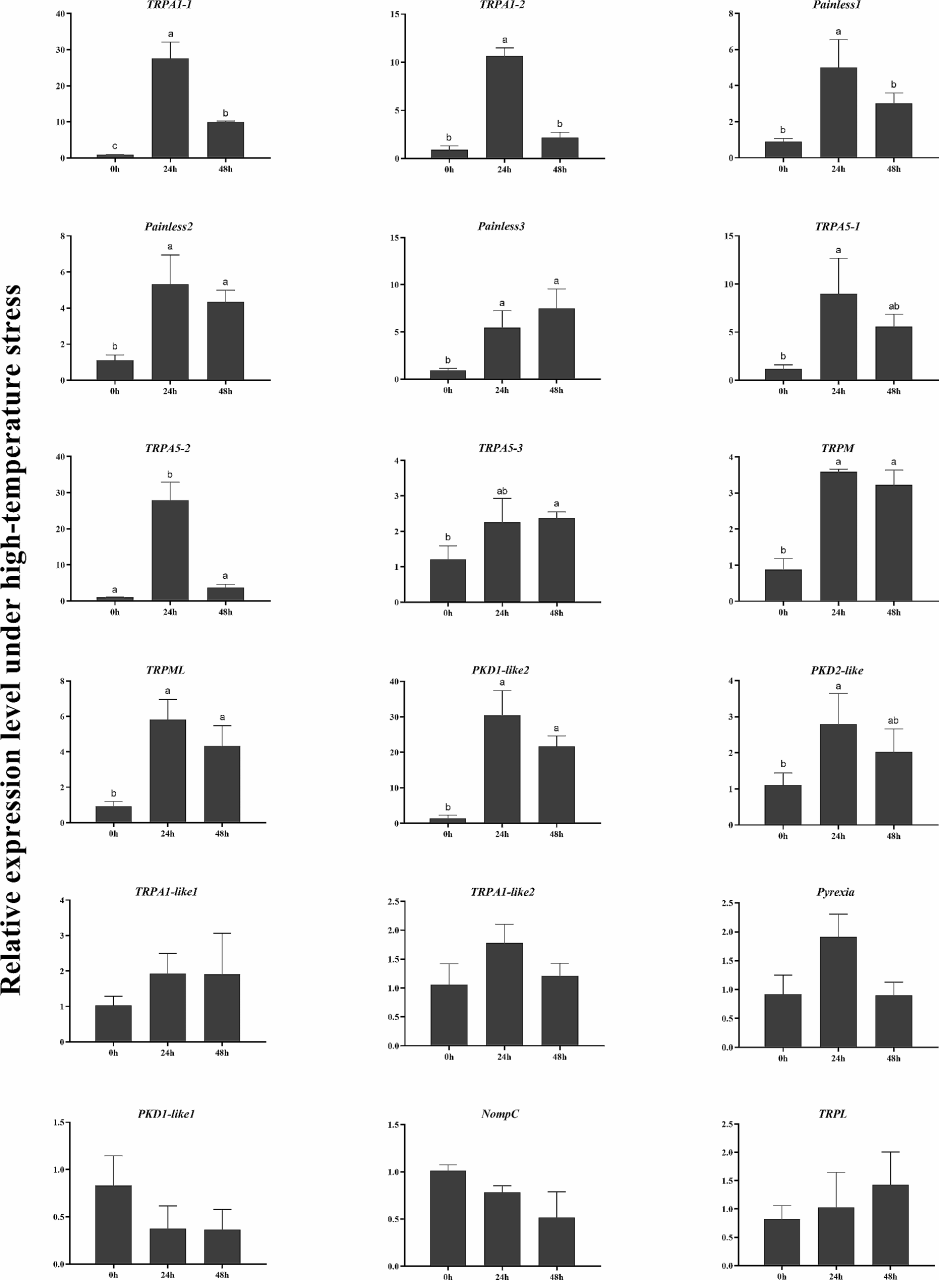
Relative expression of *TRP* genes under high-temperature stress in *P. trituberculatus*. Data are presented as mean ± SD (*n* = 3). Significant differences among groups are indicated by different letter labels (one-way ANOVA, followed by post hoc Tukey’s multiple-group comparison, *P* < 0.05).

Temperature challenge experiments were undertaken to investigate the responsiveness of identified *TRP* genes to the acute temperature change. The transcript levels of *TRPs* expressed in neural tissues and sensory structures were detected (Figs. 5 and 6). The results showed that 12 *TRPs* were significantly induced in both 18℃ and 34℃ groups, which include *PtriTRPA1-1*, *PtriTRPA1-2*, *PtriTRPA5-1*, *PtriTRPA5-2*, *PtriTRPA5-3*, *PtriPainless-1*, *PtriPainless-2*, *PtriPainless-3*, *PtriPKD1-like2*, *PtriPKD2-like*, *PtriTRPML*, and *PtriTRPM*. On the contrary, *PtriTRPA1-like1*, *PtriTRPA1-like2*, and *PtriPyrexia* showed no obvious change in either group. The other three *TRPs* including *PtriPKD1-like1*, *PtriTRPL*, and *PtriNompC* were found down-regulated in the low temperature group.

## Discussion

A total of 26 *TRP* genes were identified from the transcriptome of *P. trituberculatus*. These genes belonged to seven subfamilies, thirteen of which were in the TRPA subfamily, four in TRPC, four in TRPP, two in TRPML, and one each in TRPN, TRPM and TRPV. Some of the TRP members identified in insects were not found in this study, such as waterwitch, HsTRPA and TRPApw in the TRPA subfamily and Nanchung in the TRPV subfamily [28]. Since homologs of TRPApw and Nanchung have been reported in *P. argus* and *C. sapidus* [24], their absence in the present study may be related to the depth of transcriptome sequencing. To date, no waterwitch and HsTRPA homologue were reported in crustaceans and whether they are insect-specific requires more investigations.

TRPA subfamily is the most abundant TRP channel identified in *P. trituberculatus*. Two *PtriTRPA1* sequences were identified, which are also present in *C. sapidus* and *P. clarkia* [24], compared to only one *TRPA1* in insects. Crustacean TRPA1-2 clustered in a branch with insect TRPA1, indicating that they might be orthologues. Five *Painless* genes were identified, compared to two *Painless* genes in *C. sapidus* and *P. argus*, and one in *Drosophila* [24]. The novel PtriPainless-5 was clustered with crustacean Painless-2s, whereas Painless-3 and 4 form a new branch. In addition, one Pyrexia was identified, which was clustered with insects and *D. pulex* Pyrexia, but in a separate branch. Indeed, our phylogenetic tree showed some confusion in the nomenclature of TRPA subfamily at current time. As with many other crustacean TRP nomenclatures, the TRPs in this study were named mainly according to the sequence with the highest homology, but the accuracy needs to be further confirmed.

The TRPA subfamily has been characterized as temperature-sensitive TRP channels in many species [13]. The sole member of mammalian TRPA subfamily, TRPA1, has been shown as an intrinsic bidirectional temperature sensor in human, and a cold receptor in rodents [13, 29, 30]. Of the two *TRPA1* genes in zebrafish, the TRPA1a is specialized for chemical sensing, whereas TRPA1b responds to cold and heat stimuli [31]. Arthropods have more members in TRPA subfamily, among which TRPA1, Pyrexia, and Painless are the most well described thermosensors [16, 19, 32]. Our results suggest that the *P. trituberculatus* TRPA subfamily genes may also be involved in temperature-sensing, as most of the identified *TRPA* subfamily genes had elevated expression levels in response to the acute temperature stress. However, the induction in *PtriTRPA1* expression in both high and low temperature treatments differs from the observations in *P. clarkii* and *E. sinensis*: *TRPA1* was only induced by high temperature in the former, while by low temperature in the latter [25, 26].

It is also noteworthy that Pyrexia has been identified as a temperature-associated TRP channel in *Drosophila* [16], but was unresponsive to temperature stimuli in the present study. This may be due to its absence in the antennae or neural tissues of *P. trituberculatus*. As essential sensory organs in crustaceans, antennae are strong candidates for detecting chemical and mechanical stimuli, as well as other stimuli such as temperature and salinity [33, 34]. Indeed, a majority of the tested *TRP* genes were expressed in the antennae of *P. trituberculatus*, suggesting their closely involvements in sensing environmental changes. On the other hand, it was found that the two *TRPA1-like* genes of *P. trituberculatus* were present in antennae and nerve tissues, but not sensitive to acute temperature stress, thus may have the function of sensing other mechanical or chemical stimuli.

Four members were identified in the classical TRPC subfamily, designated as PtriTRPC-1, PtriTRPC-2, PtriTRPL, and PtriTRPgamma. In contrast to the broad tissue distribution of the TRPA subfamily in *P. trituberculatus*, the obtained *TRPC* genes showed rather tissue-specific expression. In particular, they are expressed in either the ovaries or the testis, which is consistent with the reproductive roles of TRPC ion channels in mammals and insects [35, 36]. In addition, the highest expression of *PtriTRPL* was observed in the eyestalk, which would be reminiscent of the characterized function of *Drosophila* TRPL in phototransduction [37]. Although the TRPC family genes from vertebrates and invertebrates are not all orthologous, the presence of *PtriTRPgammma* in heart and intestine conforms with the tissue distribution of mammalian *TRPC* genes, which can also be detected in heart and small intestine [38, 39]. In both mammals and insects, the *TRPC* family genes have been proposed to be involved in sensing of low temperatures [15, 38, 40]. In *Drosophila* larval, TRP and TRPL are required for their cool avoidance [15]. Interestingly, unlike those cold-sensitive *TRPs* are induced by low temperature, the expression of *PtriTRPL* was down-regulated under low temperature stress.

The reduced expression by low temperature was also observed for *PtriNompC*, a TRPN member. NompC has been identified as a mechanotransduction channel in *Drosophila*, but it also has a thermosensory function [41–43]. *NompC* is expressed in the cold-activated Class III multidendritic sensory neurons of *Drosophila* larval, and is required for the full-body contraction induced by near-freezing temperatures [14]. Paradoxically, while the paper also revealed that TRPM and PKD-2 have the same function with NompC, orthologues of these two genes in *P. trituberculatus* showed an up-regulated expression pattern when treated by low temperature. As mentioned above, the directionality of a TRP channel for sensing temperature may vary among species, the involvements of PtriTRPL and PtriNompC in cold-sensing need to be explored in further depth.

Besides their sensing properties, the TRP channels have been extensively demonstrated to be implicated in various physiological processes, and are essential for the physiology of the tissues in which they are expressed [44]. It is indeed that most *TRP* genes of *P. trituberculatus* identified in the present study were widely distributed among tissues, which may indicate their diverse physiological functions [45], but those *TRPs* that are expressed in reproductive and endocrine organs may be of interest for our future studies. In addition to the TRPC members mentioned above, many other *TRP* genes are also expressed in the reproductive system. We noticed that some of these *TRPs* showed biased expression in the gonads, for instance, *PtriTRPA1-like2*, *PtriPainless-3*, and *PtriTRPL* were preferentially expressed in ovaries, whereas *PtriTRP-2*, *PtriPKD1-like1*, and *PtriPainless-1* were preferentially expressed in testis. Since several *TRPs* have been proposed as targets for sex hormones in mammals [44], the sexual regulatory role of these gonads-borne *TRPs* in crustaceans will be an intriguing topic. Also of interest are the *TRPs* expressed in the Y-organ, a pair of molting glands of crustaceans responsible for ecdysteroids synthesis [46]. Ecdysteroids act as crucial coordinators of cell proliferation, differentiation, and apoptosis during the molting and reproductive processes of arthropods [47]. Illustrating the relationship between TRP channels and the Y-organ ecdysteroidogenesis may give insight into the mechanisms by which temperatures affect molting process.

## Conclusion

In conclusion, the present study identified and characterized 26 genes encoding for the putative TRP channels from the swimming crab, *P. trituberculatus*. The obtained *TRPs* covered all seven subfamilies known for TRP proteins, but may not represent the entire number of *TRPs* in this species. It was found that the number of TRPA subfamily expanded in crustaceans when compared with insects and mammals, but a conserved role in thermo-sensing can be suggested. Orthologues of two cold sensors in insects, TRPL and NompC, were down-regulated by low temperature stress, which may suggest a different cold-sensing mechanism. In addition to the extensive presence in antennae, the candidate sensing organs in crustaceans, many of the tested *TRPs* showed widespread expression in adult tissues, particularly represented by neural, reproductive, and endocrine tissues, indicating diverse physiological functions for *P. trituberculatus TRPs*. As shown by the acute temperature stress experiments, many of the obtained *TRPs* were transcriptional sensitive to temperature changes. However, it should be emphasized that many TRP proteins often form heteromultimeric channels that consist of two or more TRP subunits [48, 49], and some channels may be activated by associating with other TRP channels or specific proteins. Therefore, using qPCR to detect temperature sensitivity does not, by itself, infers that these ion channels are functionally relevant temperature sensors [50]. Considering the absence of data from other periods or temperature stress, further validations are needed to clarify the role of TRPs in temperature sensing.

## Methods

### Experimental animals

Healthy swimming crabs (female: 150–200 g; male: 200–250 g) were purchased from the local fisheries market in Zhenhai District, Ningbo City, Zhejiang Province, China. Three female and three male crabs were randomly selected and anesthetized on ice for 20 min before dissection. The antennules (An1), antennae (An2), antennal gland(AnG), brain(Br), thoracic ganglion(TG), heart(Ht), gill(Gi), hepatopancreas(Hp), muscle(Ms), eyestalk(Es), epidermis(Ep), intestine(In), ovary(Ov), Y-organ(YO) and testis(Te) were collected, and frozen immediately in liquid nitrogen, and stored at -80 ℃ for further use.

For temperature challenge experiments, two temperature groups (34℃ and 18℃) were set up. The high and low temperatures were maintained by a thermostatic heater and chiller, respectively. During the experiment, the samples for the two temperature groups were continuously aerated and the salinity was maintained at 27 ± 1‰. Crabs at the C3 (crablet 3) stage were purchased from a crab farm that is located in Ningbo, Zhejiang Province, China. All the C3 crabs were acclimated at the temperature of 26 ± 1 ℃ for three days before the experiment. They were then transferred to chambers for the 34℃ and 18℃ groups, respectively. Samples were collected at two time points (24 and 48 h) during experiments. Three crabs were randomly selected at each time point and the surface water was gently wiped off with gauze, before they were flash-frozen in liquid nitrogen. They were ultimately stored at -80 ℃ until RNA extraction was done.

### RNA extraction and cDNA synthesis

Total RNA was extracted from different samples using the RNA-Solv® Reagent (Omega Biotek, USA), according to the manufacturer’s protocol. RNA was quantified using a NanoDrop 2000 UV Spectrophotometer (Thermo Fisher Scientific, USA). Total RNA (~ 1 μg RNA) was used for synthesizing cDNA using the HiScript® II Q RT SuperMix (+ gDNA wiper) (Vazyme, China). This was followed by storage at − 80 °C until use in further experiments.

### Bioinformatics analysis

The sequences for *P. trituberculatus* were collected from transcriptomes as previously reported (SRR13870345, SRR13870346, SRR13870347). The genome annotation file for *P. trituberculatus* (GCA_017591435.1) was downloaded from the NCBI databases.

To search for *TRP* genes in *P. trituberculatus*, the list of annotated sequences and ORF file were scanned for keywords of previously known TRP channels such as “TRPA” and conserved amino acid sequences such as “WKFAR”, respectively [51]. Subsequently, the obtained TRP sequences were re-validated using BLASTp. The conserved domains were analyzed by CD search.

(https://www.ncbi.nlm.nih.gov/Structure/cdd/wrpsb.cgi), and sequences containing TRP, ANK, ion transport, PKD, TRPM, or TRPML domains were screened as candidate genes. The open reading frames (ORFs) of those candidate gene sequences were obtained using the ORF finder webserver.

(https://www.ncbi.nlm.nih.gov/orffinder). The transmembrane domains of the TRP channel were predicted using TMHMM-2.0.

(http://www.cbs.dtu.dk/services/TMHMM-2.0/). The distribution of the *P. trituberculatus TRP* genes on the chromosomes was analyzed using the TBtools software with default parameters, according to the genome annotation file [52]. Multiple sequence alignments were done using ClustalX. Multiple alignment files were imported to the Jalview 2.11.2 software to identify conserved sequence motifs. The phylogenetic tree was constructed based on transmembrane protein sequences and using the MEGA 7.0 software with the maximum likelihood method based on the LG + G amino acid model, and bootstrapped with 1000 replications. The species sequence accession numbers and the length of sequences used are listed in Additional File 1. The tree was visualized using iTOL (https://itol.embl.de/).

### Reverse transcription-PCR

To detect the expression levels of *TRP* genes in different tissues, gene-specific primers were designed using the primer 5.0 software and synthesized by YKang Biotech (Hangzhou, China). The gene-specific primers that were designed by the primer 5.0 software are presented in Additional Information 2. RT-PCR was performed with 2 × Es Taq MasterMix (Dye) (CWBIO, China) according to the manufacturer’s instructions. β-actin was used as the internal reference. Amplifications were performed as follows: 94℃ for 3 min, followed by 35 cycles of 94℃ for 30 s, 57℃ for 30 s, and 72℃ for 30 s, with a final elongation at 72℃ for 10 min. PCR products were separated on 1.5% agarose gel electrophoresis and visualized using GelRed (Biotium).

### Quantitative real-time PCR

Quantitative Real-time PCR (qPCR) was performed to analyze the expression profiles of *TRP* genes under acute temperature stress. The specific primers that were used in this study were designed by primer 5.0 software and are presented in Additional Information 2. PCR was carried out using the Taq Pro Universal SYBR qPCR Master Mix (Vazyme, China), according to the manufacturer’s instructions. The cycling parameters were as follows: 95℃ for 3 min, followed by 40 cycles of 95℃ for 15 s, 60℃ for 30 s, 72℃ for 15 s. To confirm product specificity, a melting curve analysis of the amplified DNA was performed following amplification, at temperatures between 60 and 95 °C, with the temperature increasing at a rate of 0.15℃/s. The relative mRNA expression levels were normalized to β-actin mRNA expression. Calculations for the relative mRNA expression levels were done using the comparative Ct (2^−ΔΔCt^) method.

### Statistical analysis

All data were expressed as mean ± SD (standard deviation). The statistical differences were analyzed using one-way analysis of variance (ANOVA), followed by Tukey’s multiple-group comparison test (SPSS 22.0 software). Significant differences were accepted at *p* < 0.05.

### Electronic supplementary material

Below is the link to the electronic supplementary material.

**Supplementary Material 1: Supplementary Table 1** The species sequence accession numbers

**Supplementary Material 2: Supplementary Table 2** Primers used for RT-PCR. **Supplementary Table 3** Primers used for qPCR

**Supplementary Material 3:** Full-length gels which have been cropped in the main text

## Data Availability

All data generated or analyzed during this study are included in this published article and its supplementary information files.

## Abbreviations

PKD: Polycystin or Polycystic Kidney Disease
TRP: Transient Receptor Potential channels
TRPA: Transient Receptor Potential Ankyrin
TRPC: Transient Receptor Potential Canonical
TRPM: Transient Receptor Potential Melastatin
TRPML: Transient Receptor Potential Mucolipin
TRPN: NompC, or no mechanoreceptor potential C
TRPP: Transient Receptor Potential Polycystic
TRPS: Transient Receptor Potential Soromelastatin
TRPV: Transient Receptor Potential Vanilloid
TRPVL: Transient Receptor Potential Vanilloid-like
