# Supplementary material for: Identification of transient receptor potential channel genes from the swimming crab, Portunus Trituberculatus, and their expression profiles under acute temperature stress
Source: BMC Genomics. 2024 Jan 17;25:72. doi: 10.1186/s12864-024-09973-x (PMC10795286; doi:10.1186/s12864-024-09973-x)
Supplement: Supplementary file 2 — Supplementary Material 2: Supplementary Table 2 Primers used for RT-PCR. Supplementary Table 3 Primers used for qPCR [file 12864_2024_9973_MOESM2_ESM.docx]

Supplementary Table 2 Primers used for RT-PCR.

| TRP genes | Primer sequences（5^’^-3^’^） |
| --- | --- |
| TRPA-1 | F-CGAGGTTGGCGGAGCAT  R-GGCAAGGGAACTGATCTGG |
| TRPA1-2 | F-ACGCCTGAATCTTCTCAACCAT  R-ATTACCCACTTTCCAGCCACTAC |
| TRPA1-like1 | F-GCTCTGCTCCCCACCTCCA  R-ATCATCCACACCATCACCTGC |
| TRPA1-like2 | F-GGCTCCTACTCATCAGCAAGC  R-AGTCCCCAAGCAACCATACAAT |
| TRPA5-1 | F-CCTCCAGCGGCAGGGTAAGA  R-GGCGGTCACGATCATATTTGATTT |
| TRPA5-2 | F-GGATACCGTGTTTGACGAGTGC  R-TGGGTGGCGTGGTTAGGG |
| TRPA5-3 | F-AGTTTTCTTTGGCTGGGTTGA  R-TAATCGCCGTGTTTTCGTTC |
| Painless1 | F-TGTCTGAGGTTCCTGAGTGGTTC  R-TCTGTTGCTGGTACAAAAGTTCC |
| Painless2 | F-CAAGCAGGGGAAGCAAATC  R-TTCAGCACGAACACGAACC |
| Painless3 | F-GGAAACGAGAACGAGGAGGAAG  R-GTACGCAAATACACTCAGCAACCC |
| Pyrexia | F-CGTTCTTTCTTCGTGTTGAACTTG  R-GGATCTAATAGGCGTCGGGTGT |
| TRPL | F-GTCAAGTTCTTCTTCATTTACACCC  R-AGCTGGACATCATAGCGATCAG |
| TRP-1 | F-GCTTATGTTTGGTTCGTACTCCG  R-TGTCCCGCTGTTCCGCA |
| TRP-2 | F-GAAATTCGCTCGGTCTAAGCT  R-CTCGTCATTCCTCCTCTGCAT |
| TRPgamma | F-CCGCTCCGCTCTGCTTATG  R-CGTGATGTCCGGGGTGAA |
| Inactive | F-CAGGACGGGATAATGAACGA  R-TGGTTCCTGGCGAAGGTC |
| NompC | F-CAGTGCAGCTCTTGGATTTCTTG  R-TAACCTGTTGTCTCGCCGTCT |
| PKD2-like | F-CAAATCTGAAGGGGACAAGCA  R-TCACCAGAACGGCGTCAAT |
| PKD1-like1 | F-ATTCATACCCTCCTGGAACTTC  R-AATCCTCGGCTCACCTTAGAC |
| PKD1-like2 | F-AGGGCTTGCGGCTATCGT  R-CTCGGCGGCCTTTGTCC |
| TRPM | F-AGAGGCCCAACTGGTCAACTC  R-GTGAACAAACACAGGAAGAAAACG |
| TRPML | F-GGATACTTGGACACCTGGAATGTA  R-TGGACGAGAATGAACGGAACTT |
| β-actin | F-CGAAACCTTCAACACTCCCG  R-GGATAGCGTGAGGAAGGGCATA |

Supplementary Table 3 Primers used for qPCR.

| TRP genes | Primer sequences（5^’^-3^’^） |
| --- | --- |
| TRPA-1 | F-TGAGGCAGACGAGTGTGACGA  R-CCTTCTTGGGCAGTGGCAT |
| TRPA1-2 | F-AGATGAGCGAGCAGGAGTCAA  R-AGATGAGCGAGCAGGAGTCAA |
| TRPA1-like1 | F-GGCGGAGGCGATTTGTCTA  R-CGATTTGATGCCGTAGGTGAG |
| TRPA1-like2 | F-CTCCACTACATACTGCCATCAAAC  R-GAGTCATTCCTCCCTTACACAC |
| TRPA5-1 | F-CGCTGCTTGTCTCCAGGGTT  R-GCCGCTCTGCACTGCGTAAT |
| TRPA5-2 | F-ACCAGAAGAGCGAAGCGAATA  R-TCAGCGTAAGTGTCTCTCATCTCC |
| TRPA5-3 | F-CAACGCAACCTGCCCATTC  R-TCCAGCCTTCACAGATCGC |
| Painless1 | F-GGCAACACTGCTTTACATTATGC  R-AGCACAGATGCCTGAATACGG |
| Painless2 | F-ACCACGCAGAACGAAGATCAC  R-CCGCCTCCTGCTGGTTAT |
| Painless3 | F-CAGGGATAGTAGCGGGGATG  R-GCACGGCGTAAAACAGTAGAC |
| Pyrexia | F-GGACACACCCGACGCCTATTA  R-CACCGCTCCCTCTTGGATATT |
| TRPL | F-GCCTCCGCCCTTCAACA  R-TGCACGCATCACAGACAGGT |
| NompC | F-GCTGACAGAGATAGCAAAAATCG  R-GGCAGCGGAATGGACAATAA |
| PKD2-like | F-ACCAAGGATGACCGCAACA  R-GATGACTCGCTGGAAGTCTGG |
| PKD1-like1 | F-GCCAGTGGTTGTTGCCTATC  R-GGTAGACACGGTTCCATTACATTCAA |
| PKD1-like2 | F-GAGTAACCGAAAGCATAGCCC  R-CCTTCCCTCTCCCCTGTGT |
| TRPM | F-CTATCCCGAGCACCCTAGTTG  R-TTGAGCGGATAGTGAGAAAAGC |
| TRPML | F-CCTGCTATGGCTCTGTTTCA  R-GAGTCATATTTGGGCAGTGGAC |
| β-actin | F-CGAAACCTTCAACACTCCCG  R-GGATAGCGTGAGGAAGGGCATA |
