# Supplementary material for: Identification of transient receptor potential channel genes from the swimming crab, Portunus Trituberculatus, and their expression profiles under acute temperature stress
Source: BMC Genomics. 2024 Jan 17;25:72. doi: 10.1186/s12864-024-09973-x (PMC10795286; doi:10.1186/s12864-024-09973-x)

Full-length gels which have been cropped in the main text.

RT-PCR results in Figure 4 of the main text, red lines represent cropping lines. All gels have been run under the same experimental conditions.

1. TRPA1-1


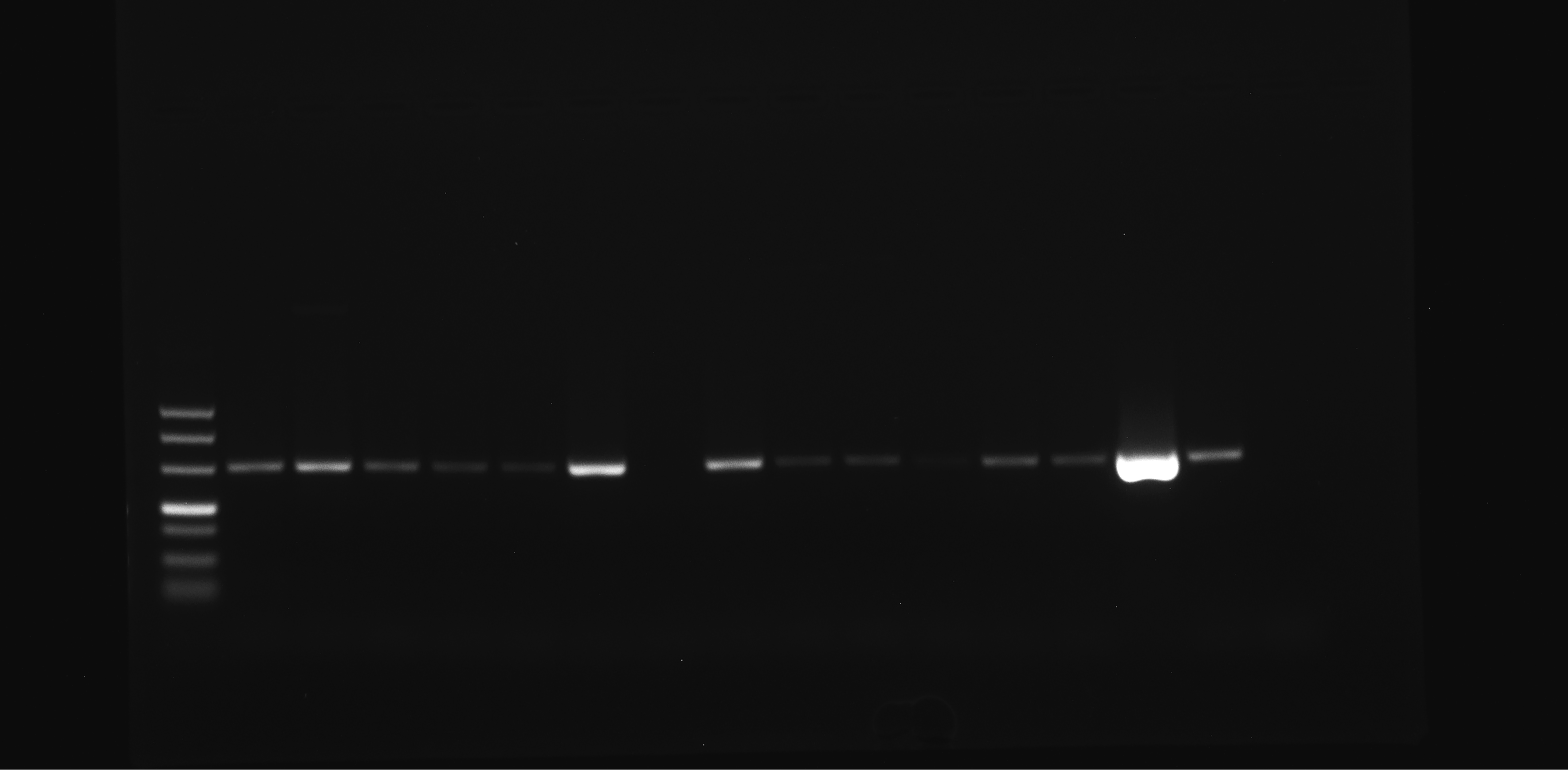


1. TRPA1-2


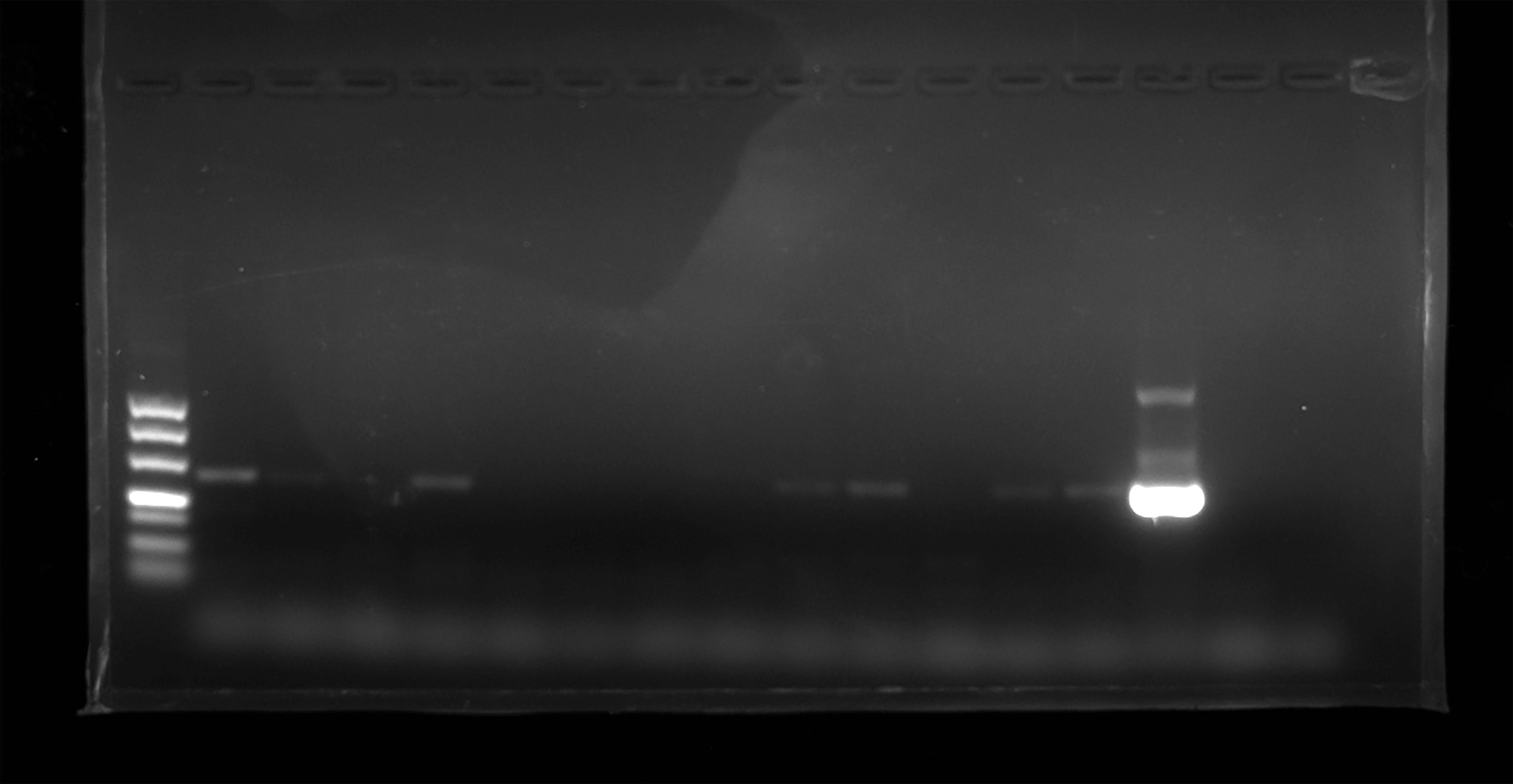


1. TRPA1-like1


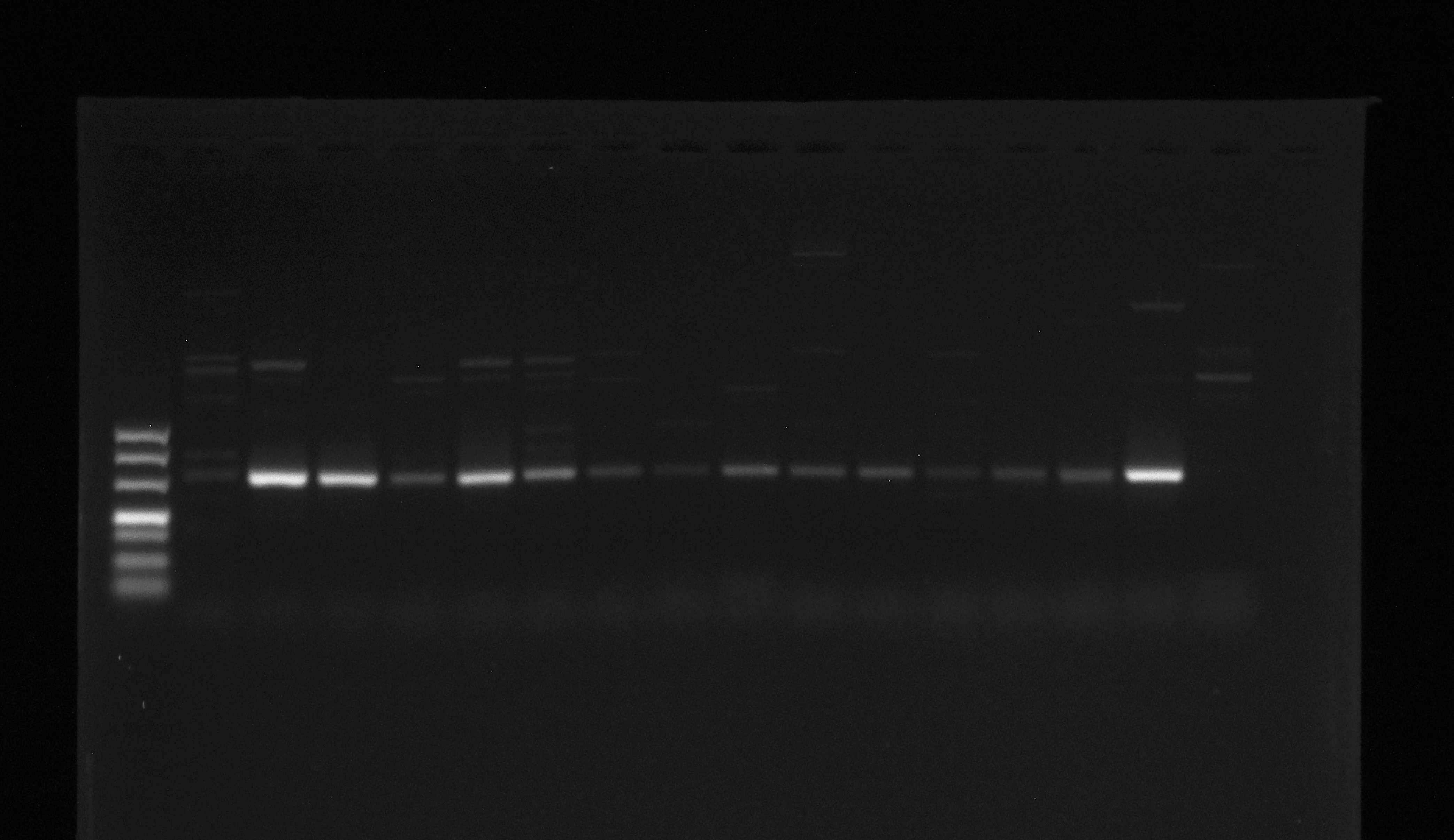


1. TRPA1-like2


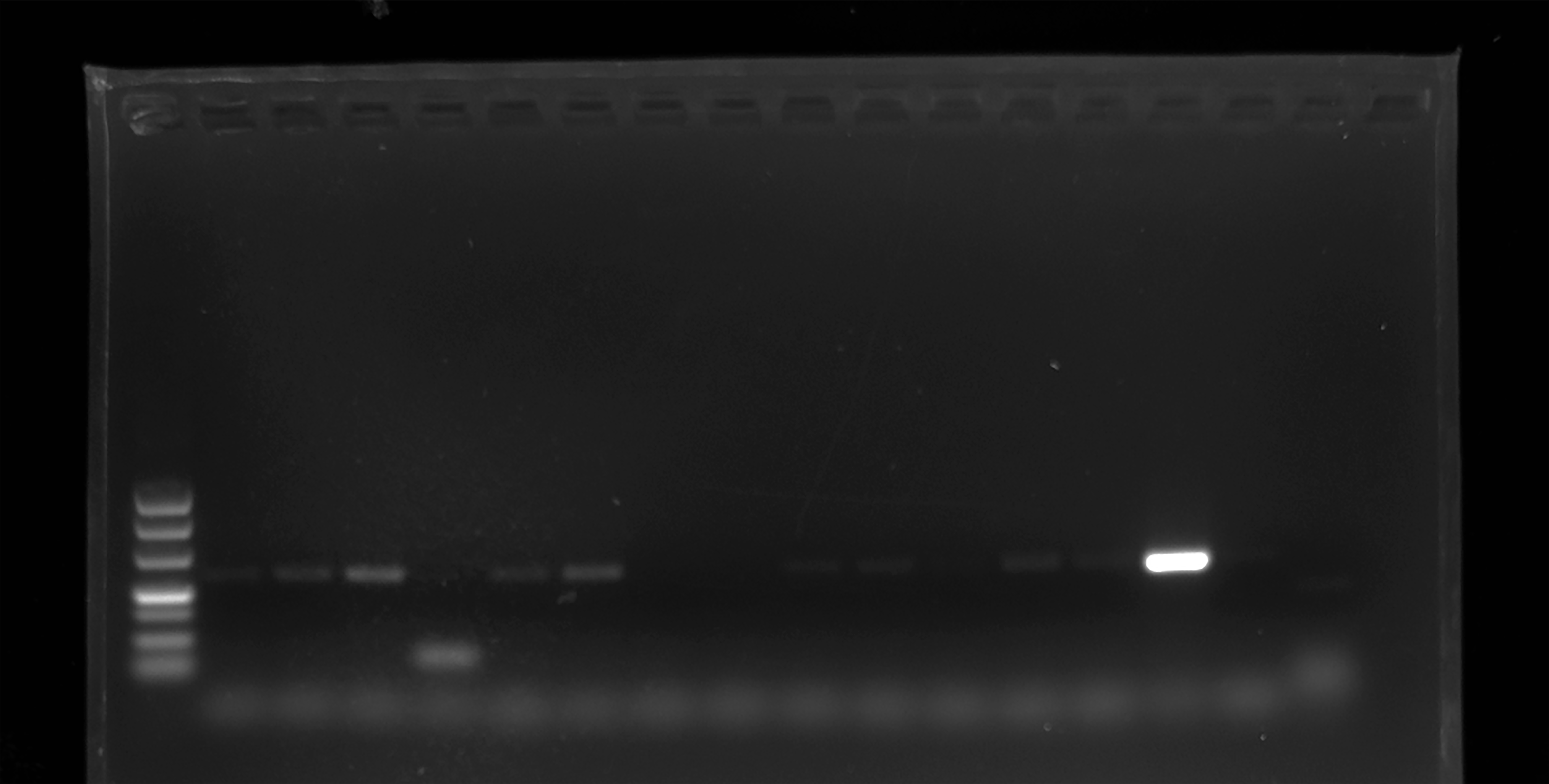


1. TRPA5-1


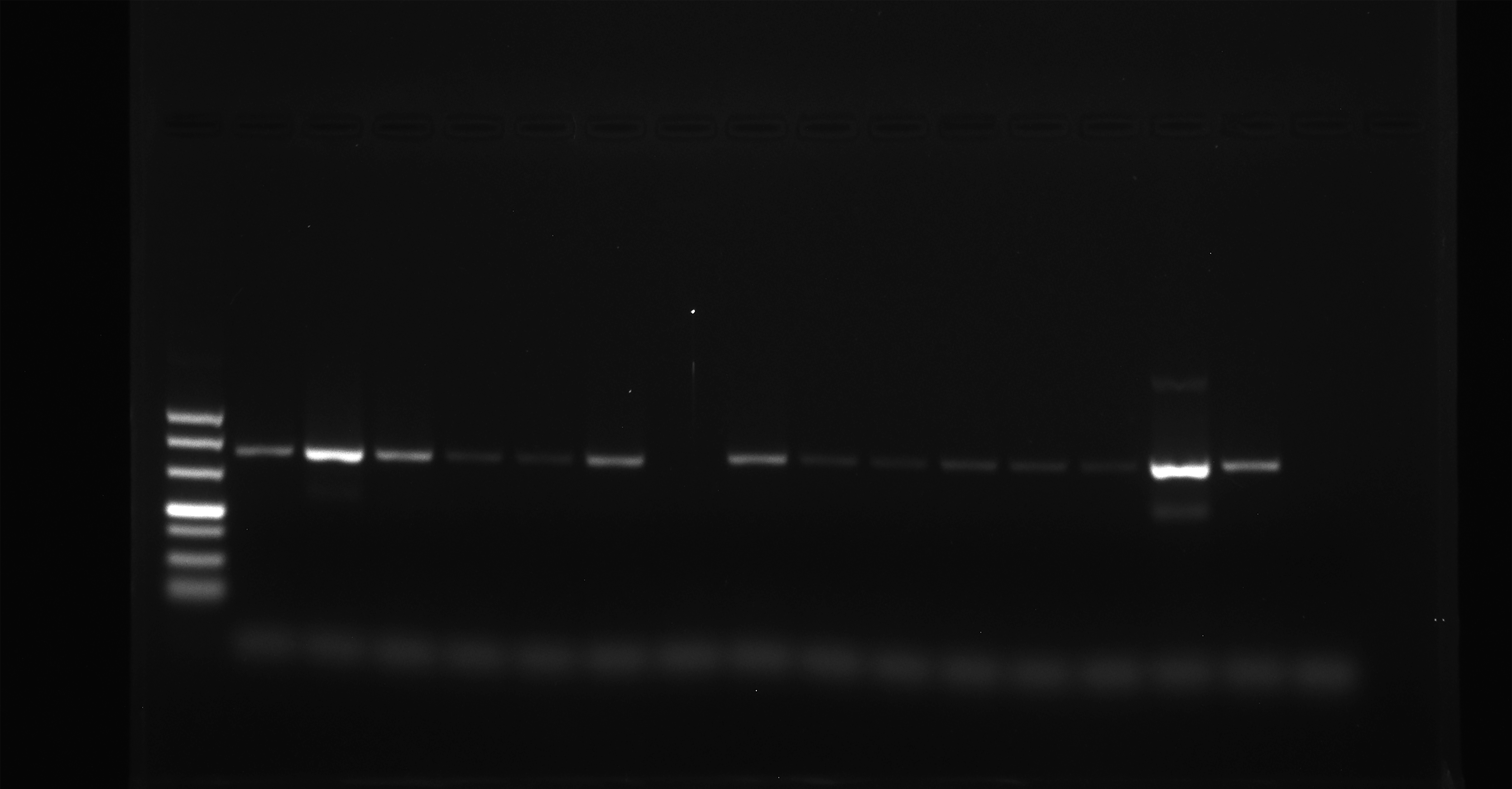


1. TRPA5-2


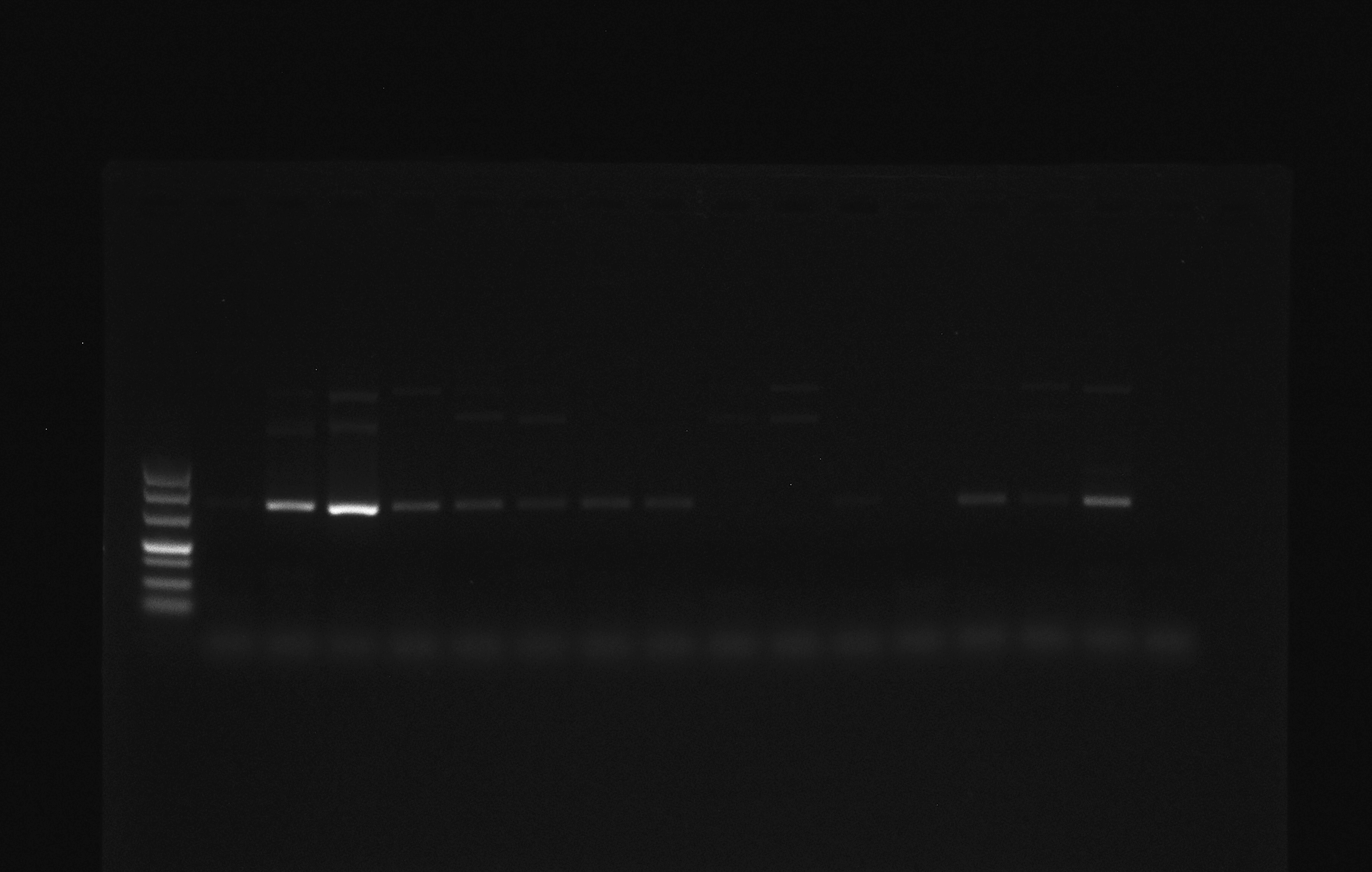


1. TRPA5-3


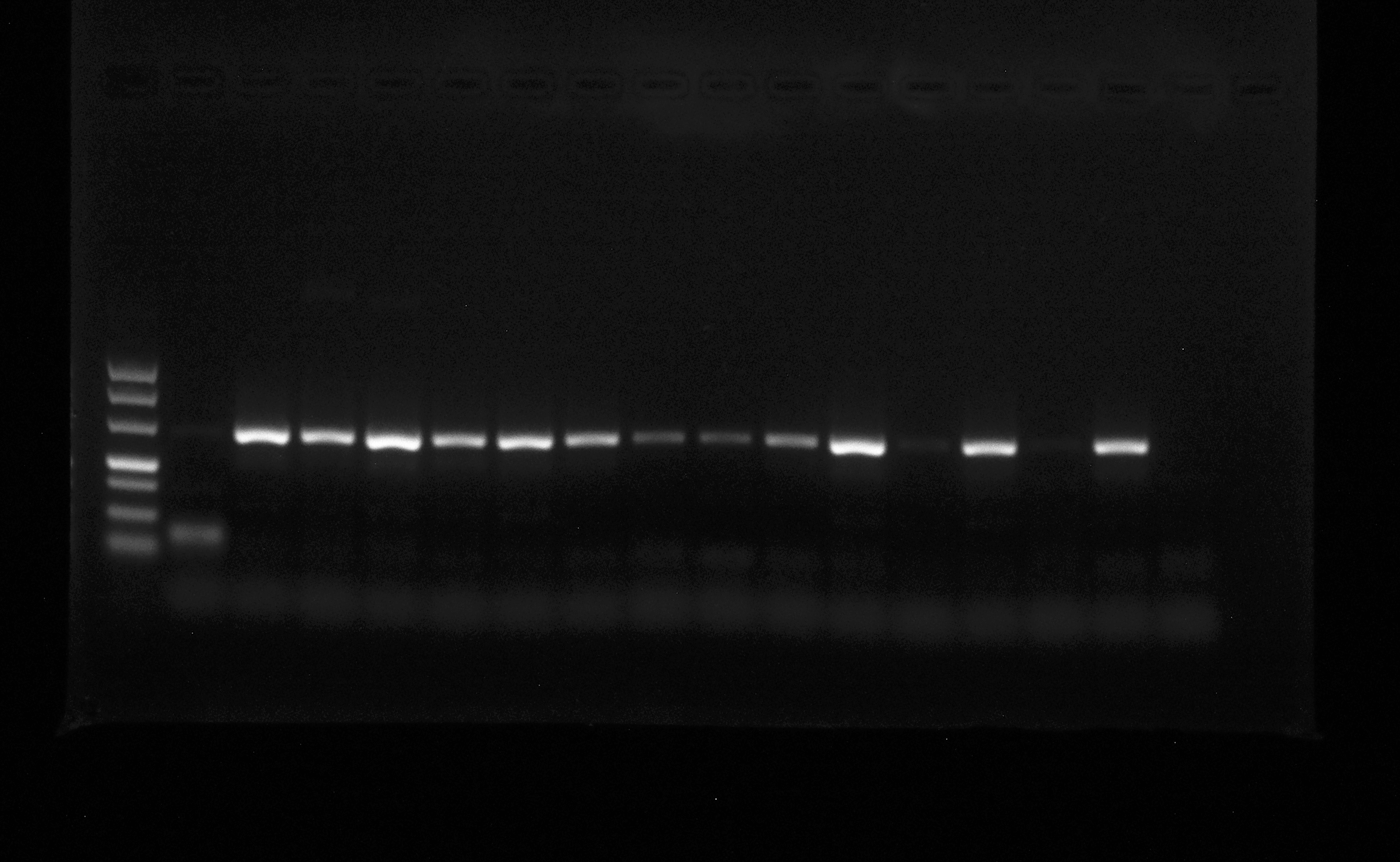


1. Painless1


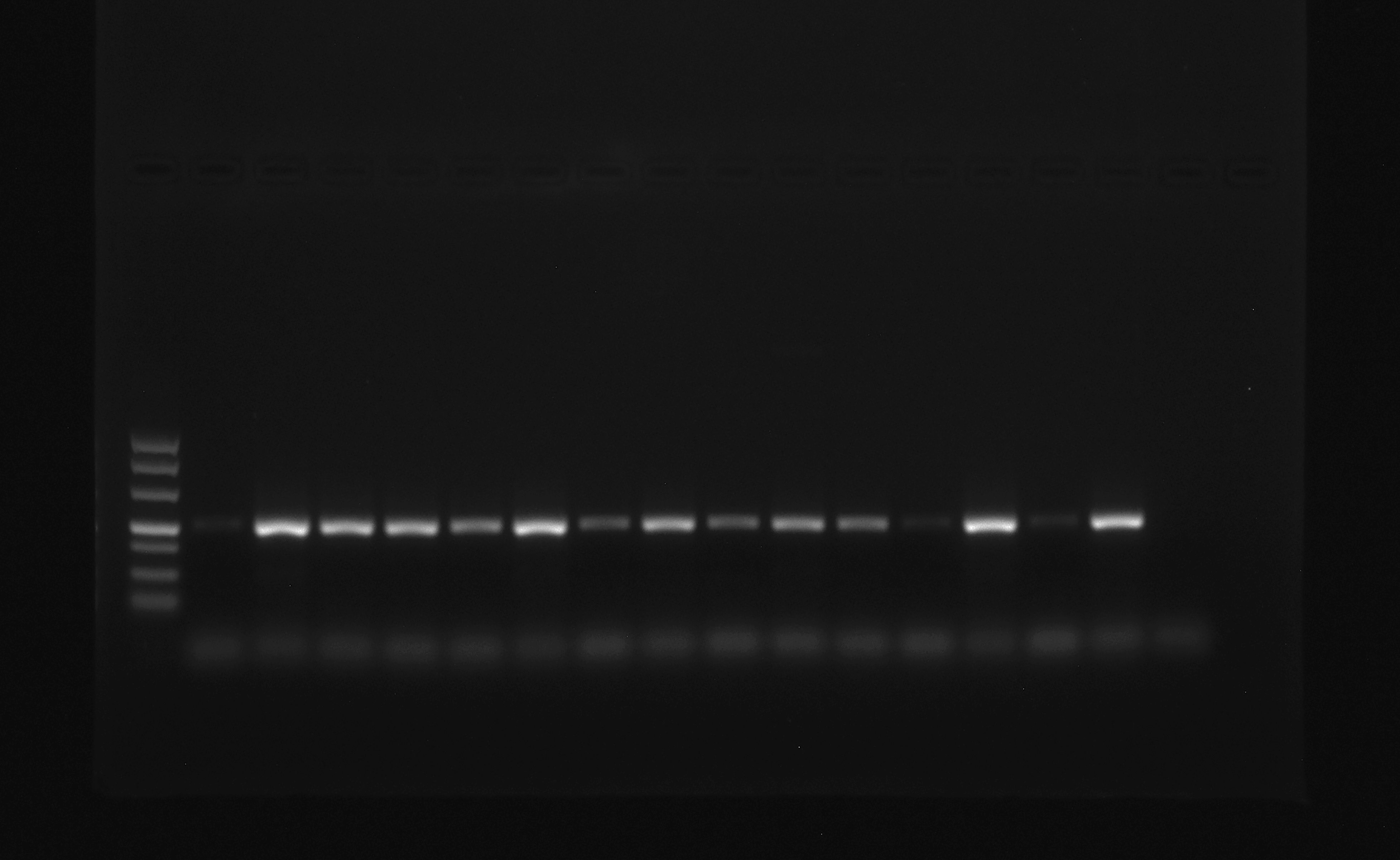


1. Painless2


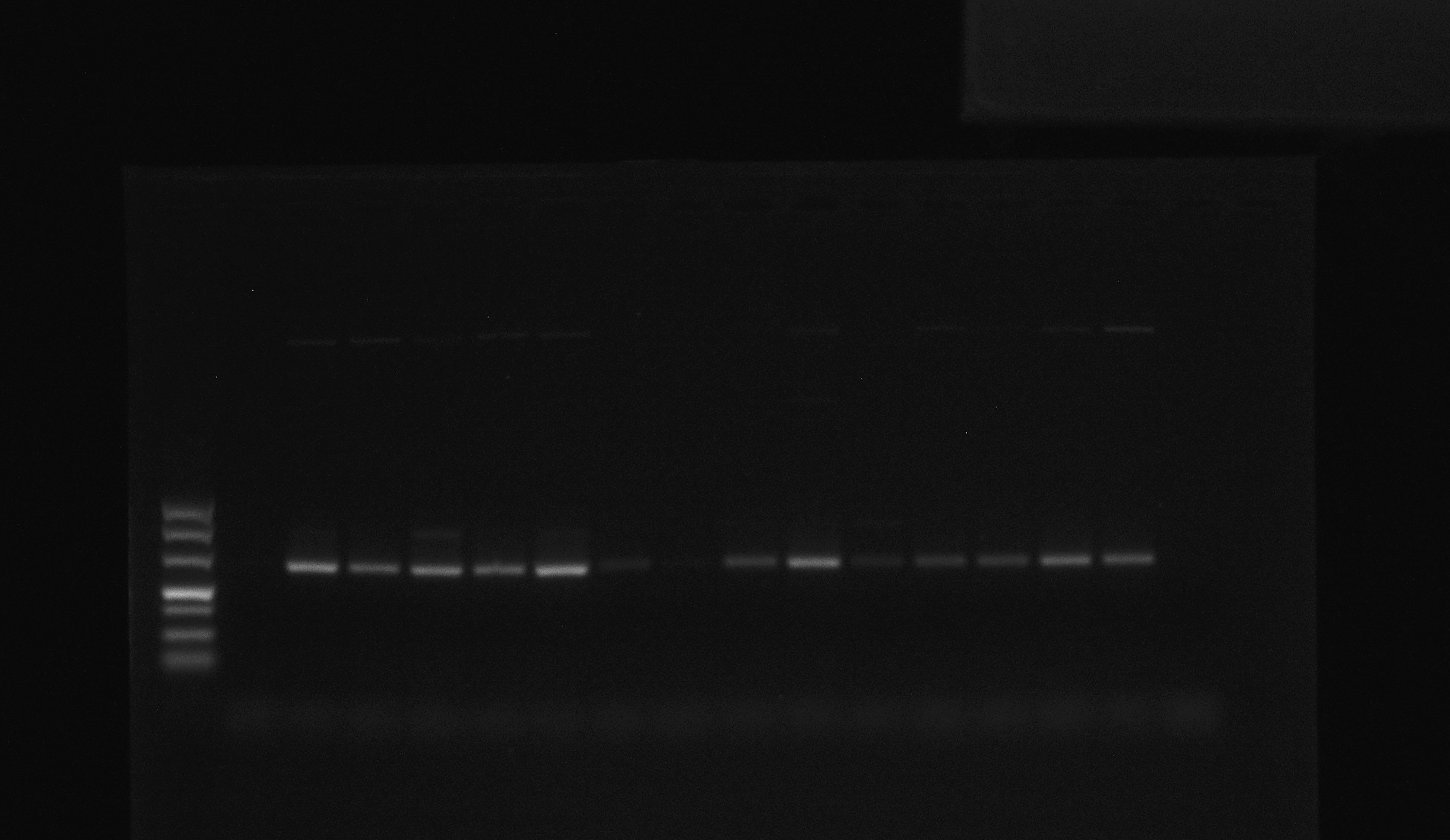


1. Painless3


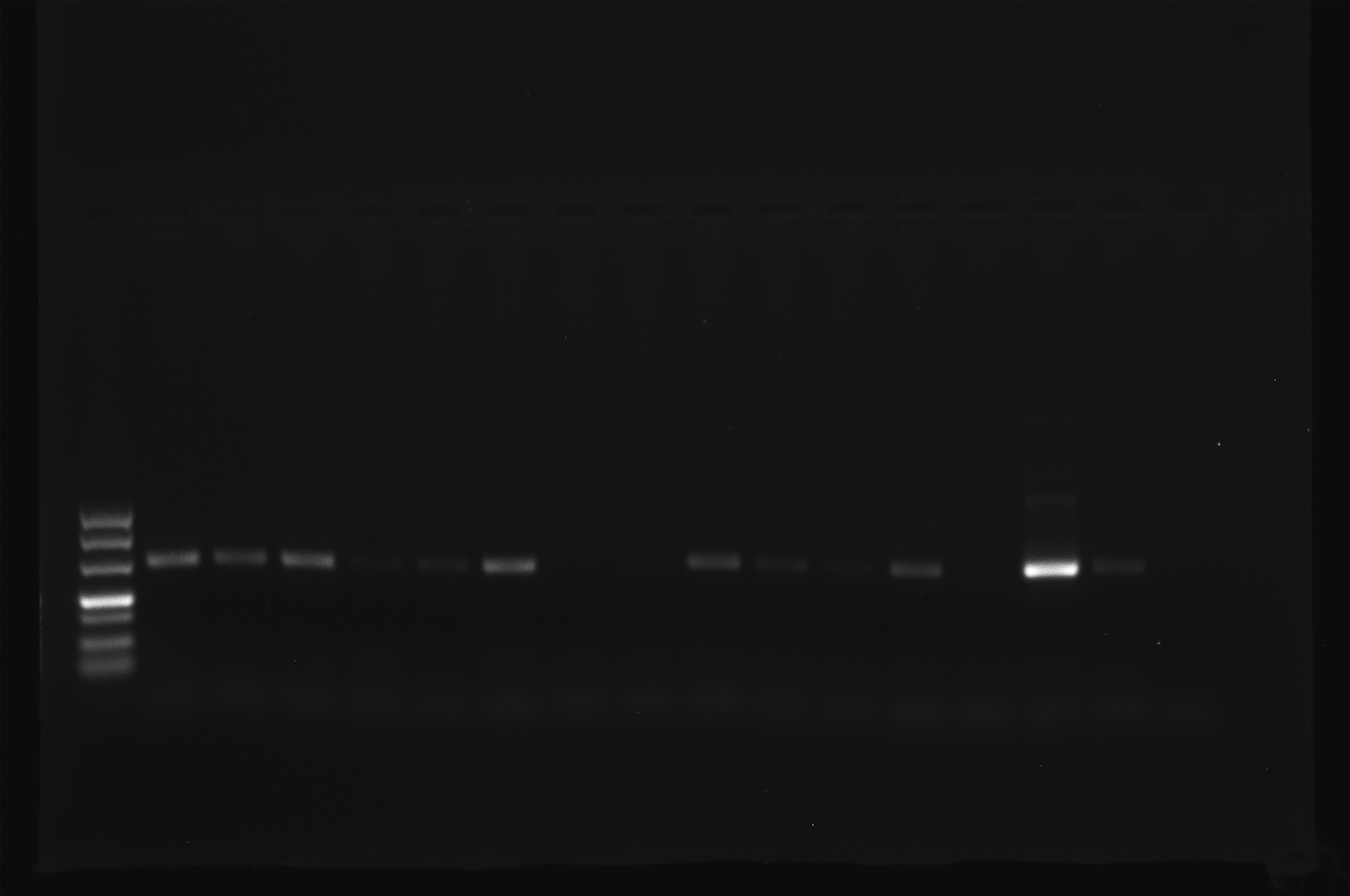


1. Pyrexia


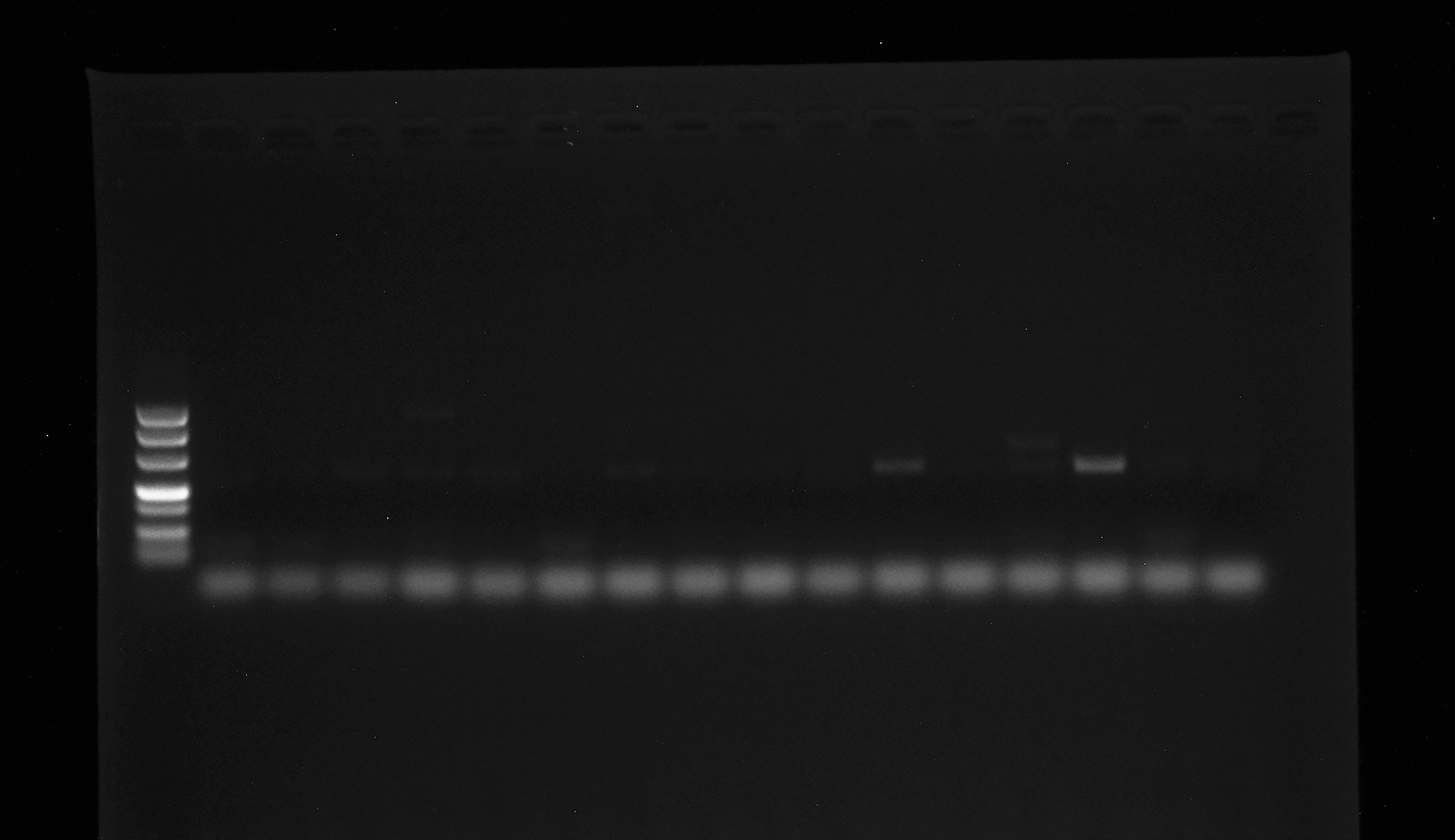


1. TRPL


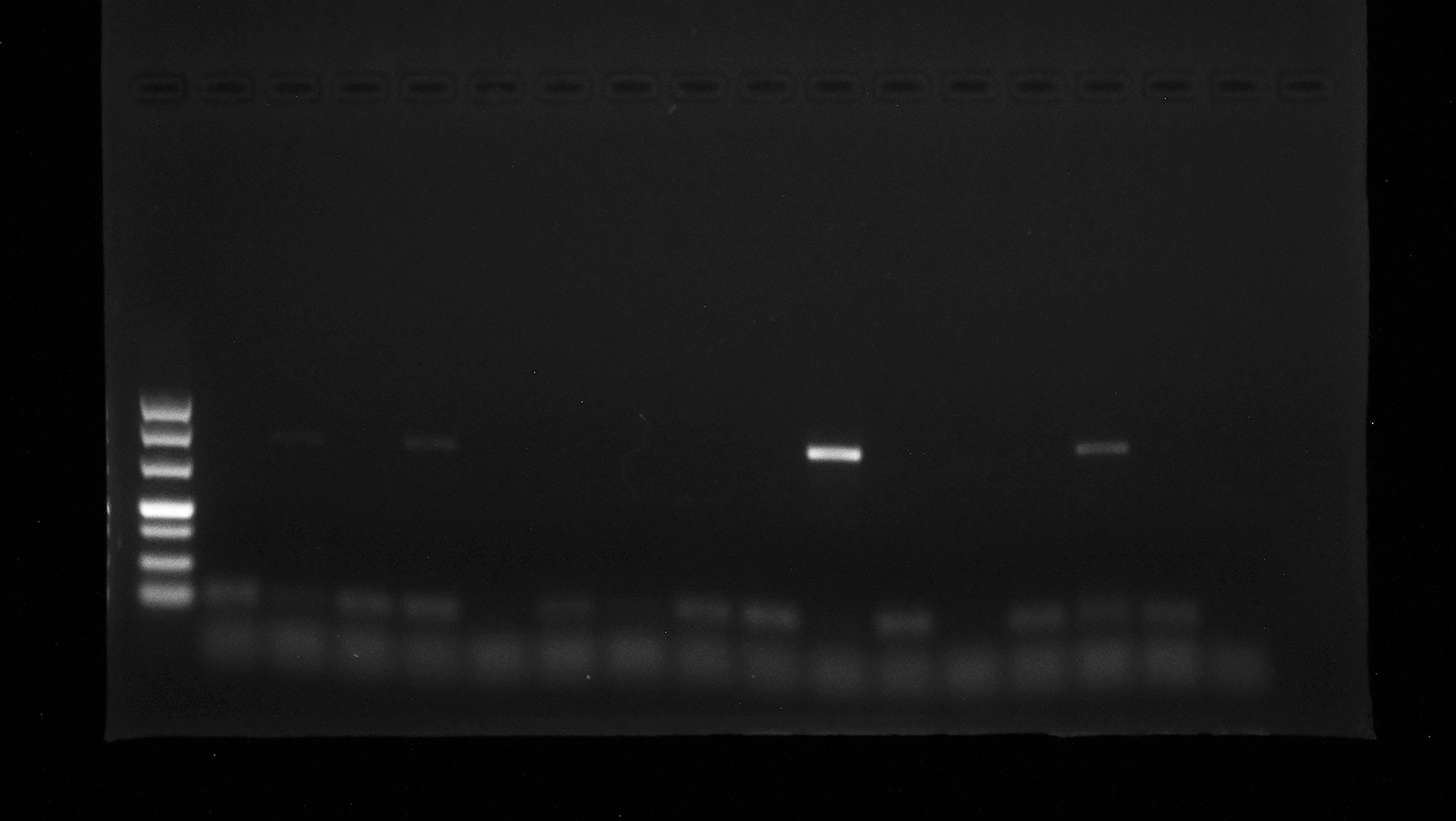


1. TRP-1


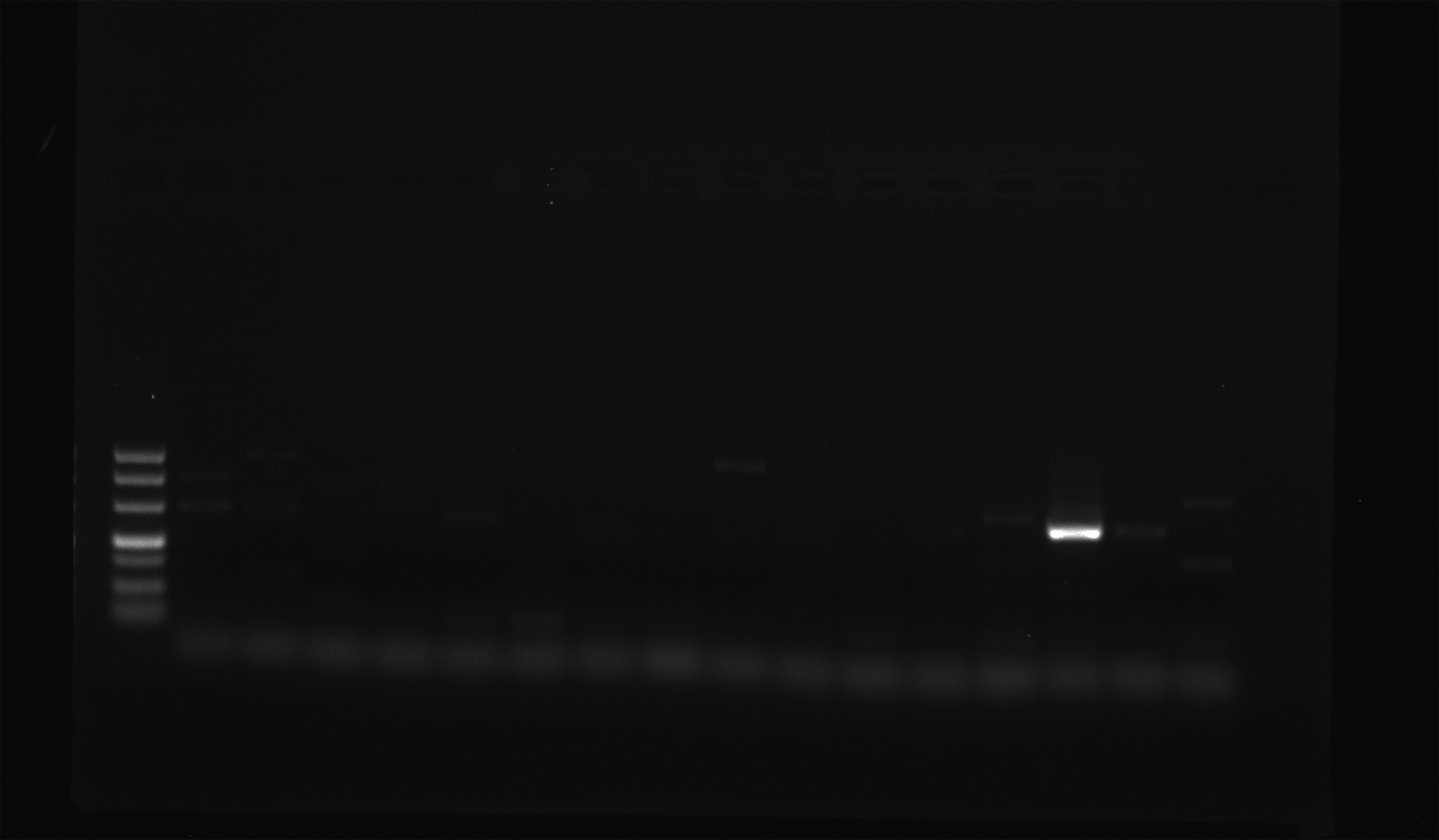


1. TRP-2


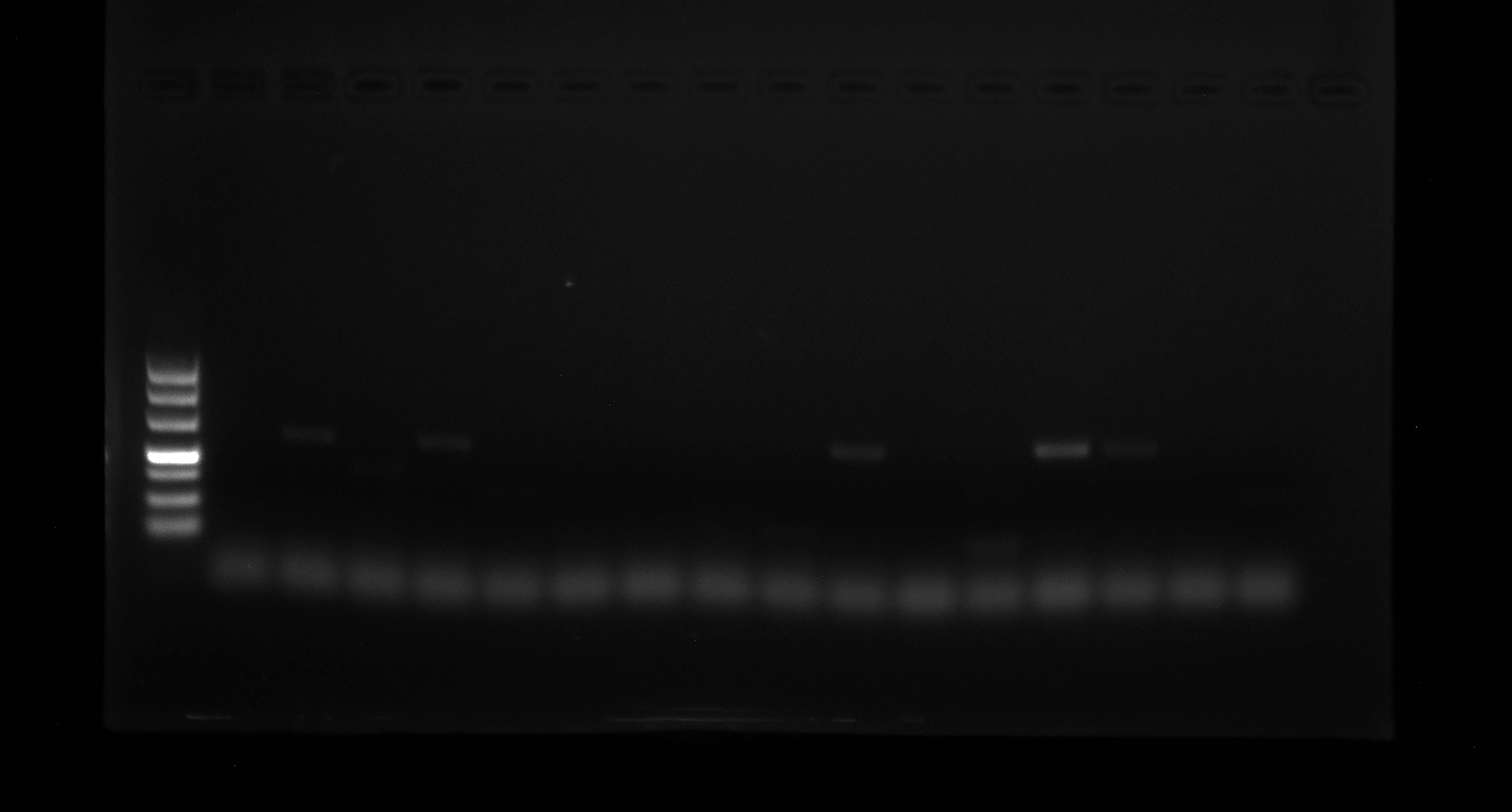


1. TRPgamma


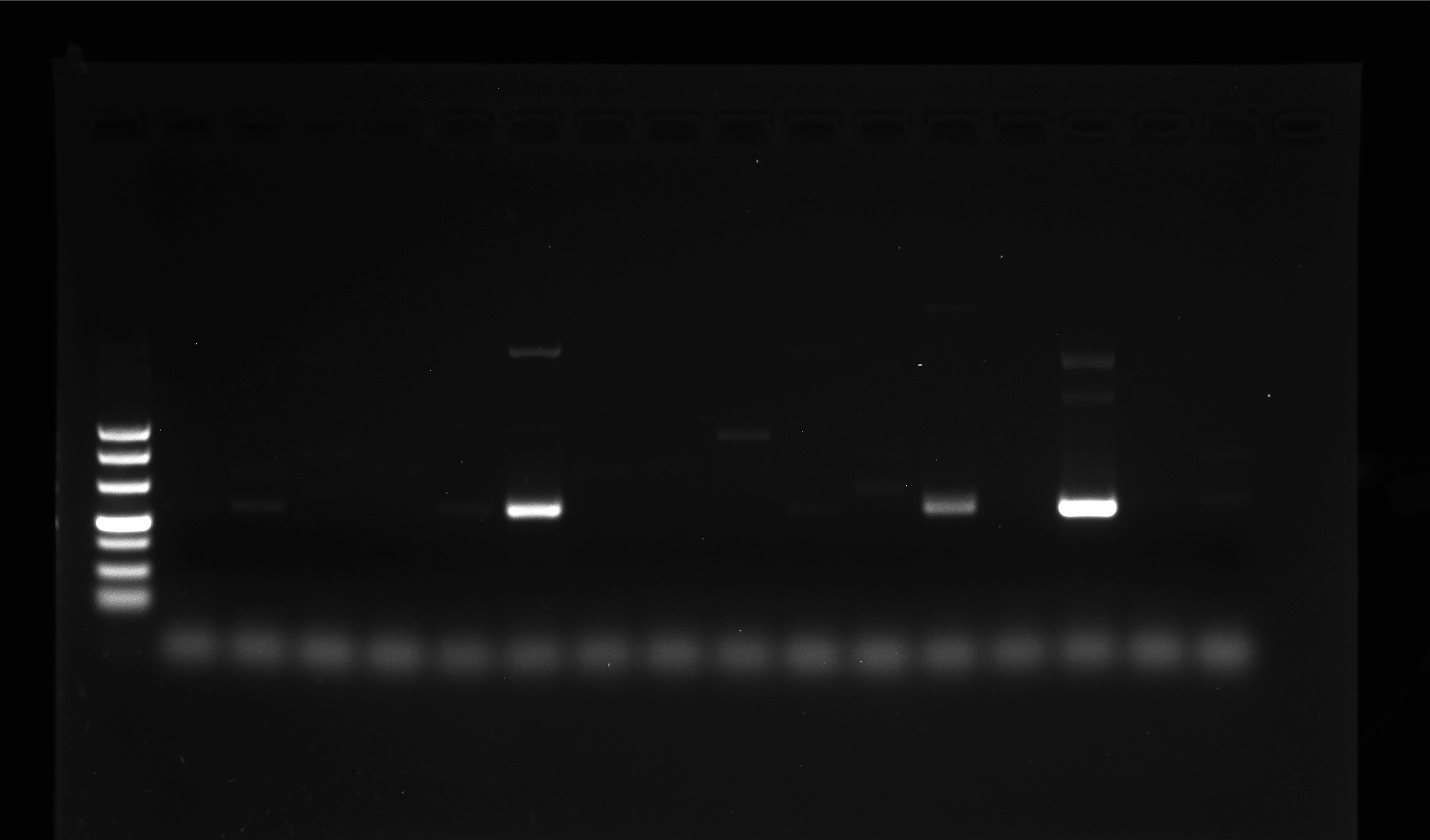


1. Inactive


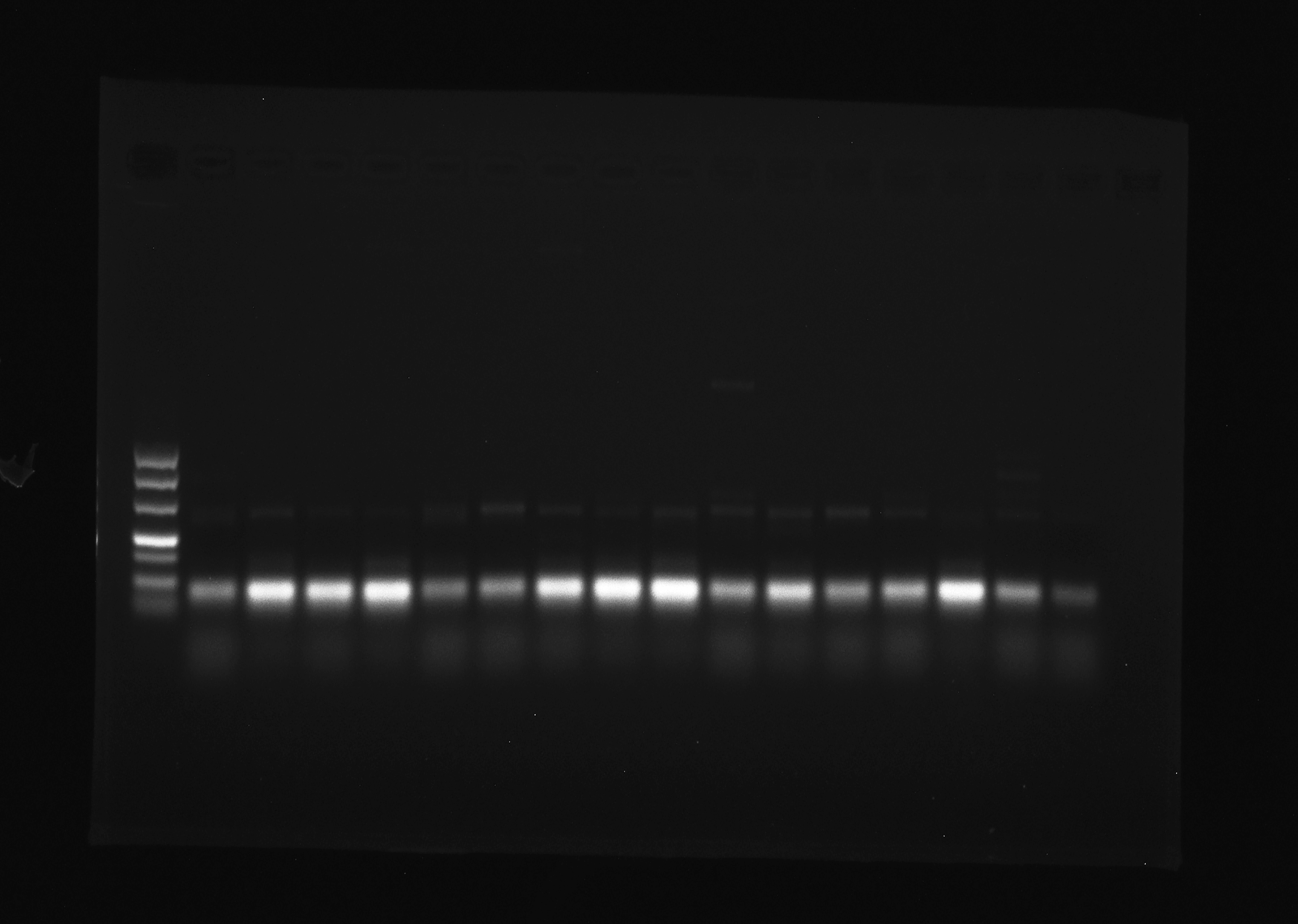


1. NompC


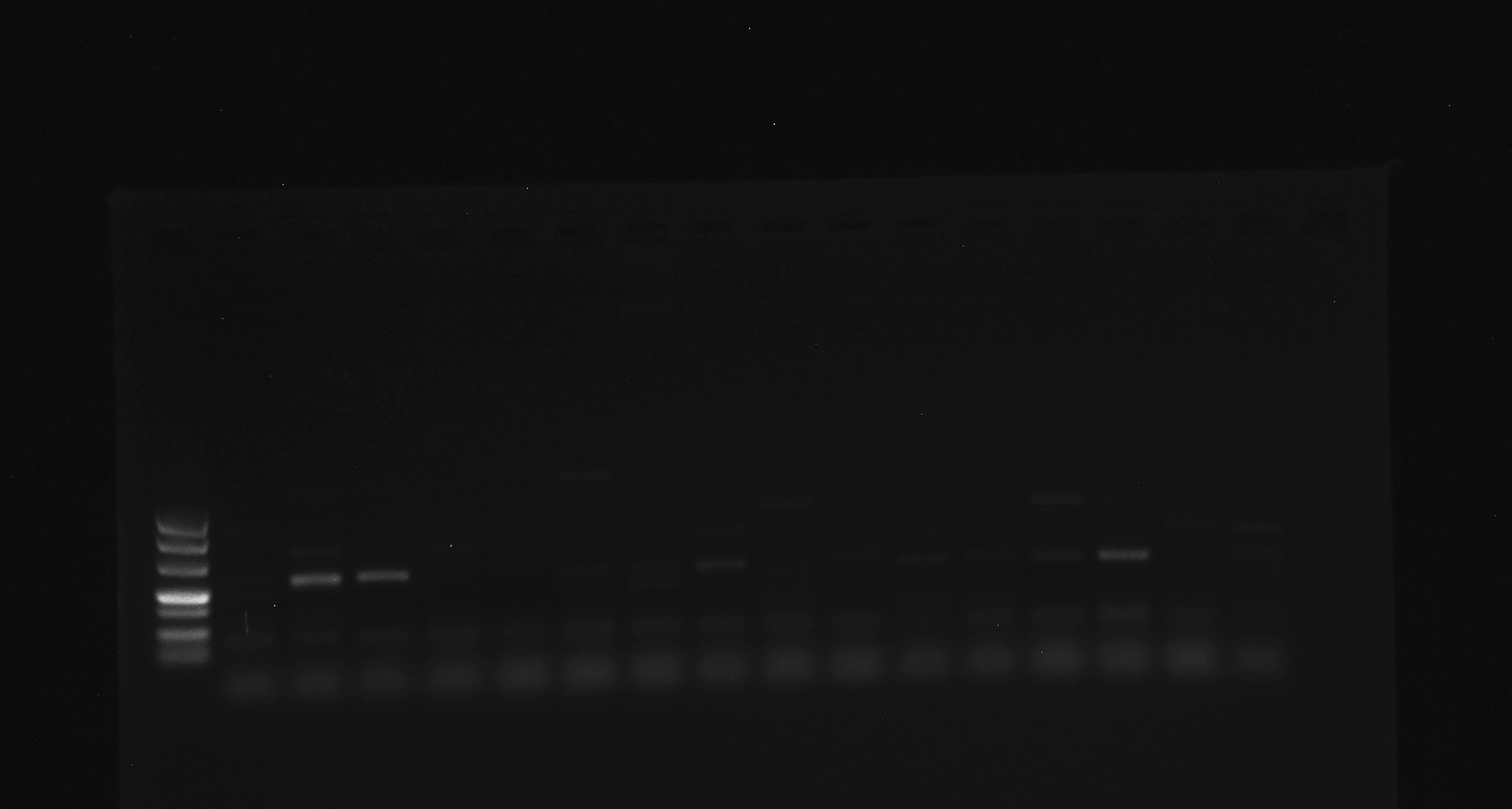


1. PKD2-like


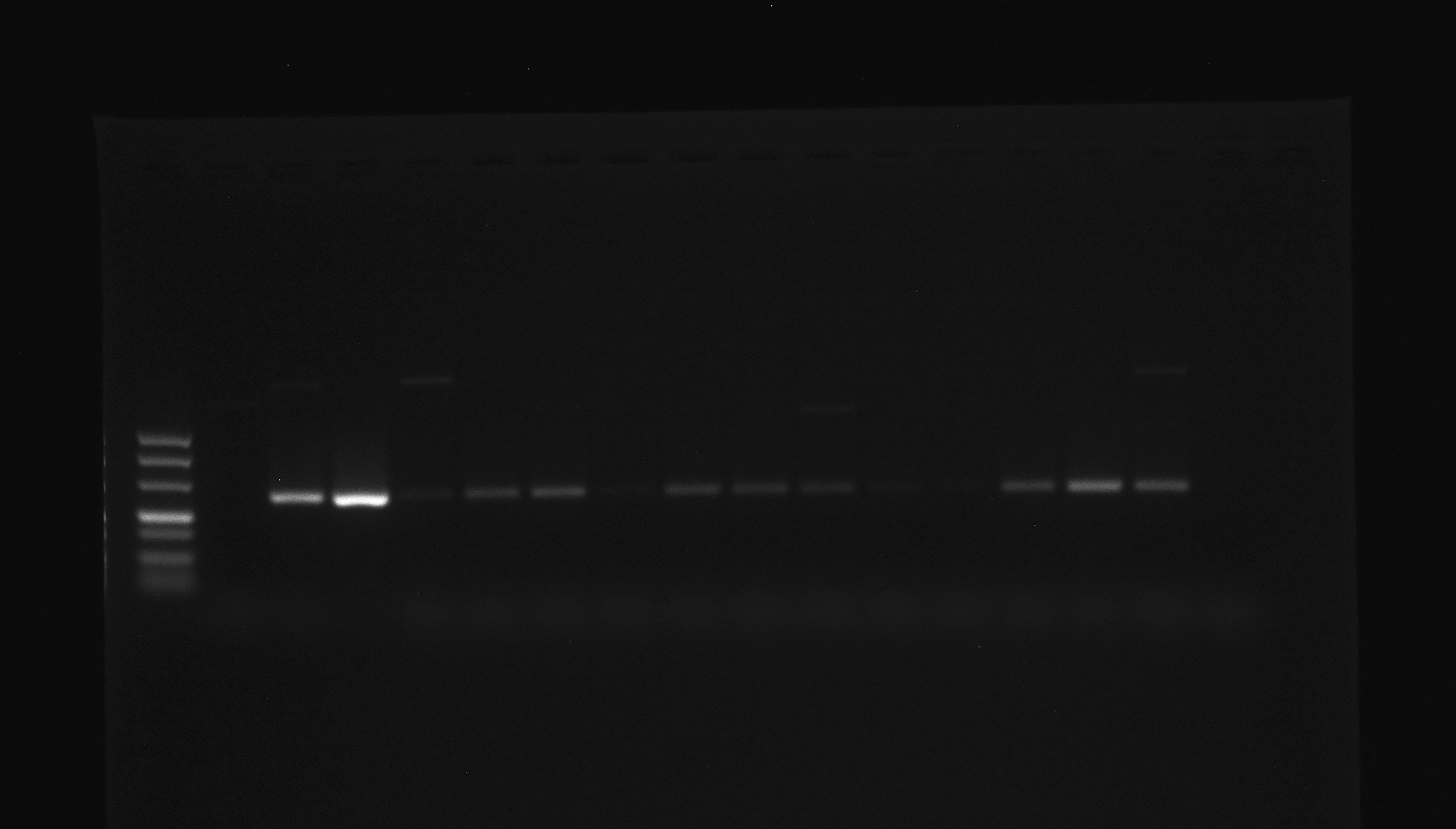


1. PKD1-like1


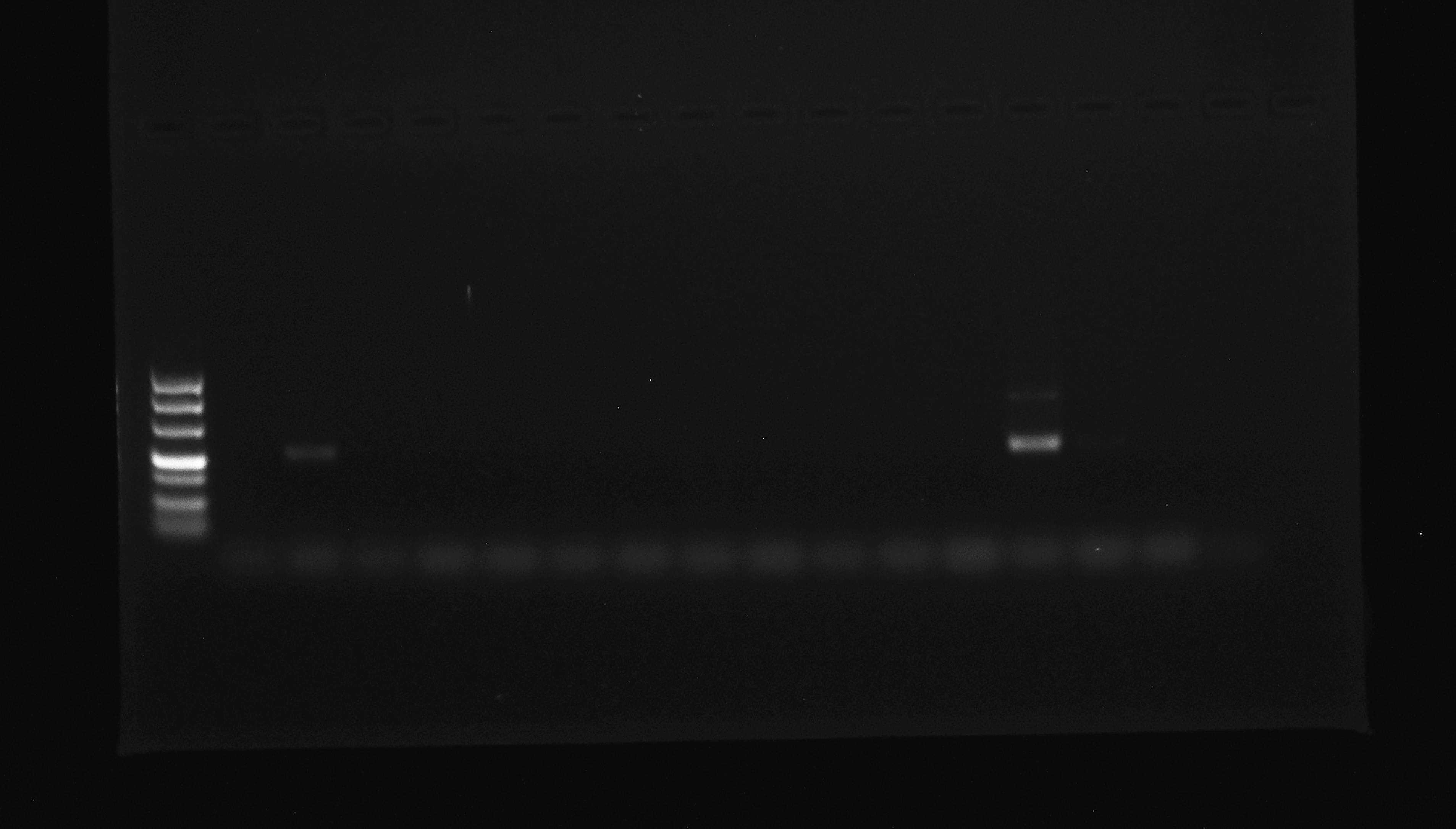


1. PKD1-like2


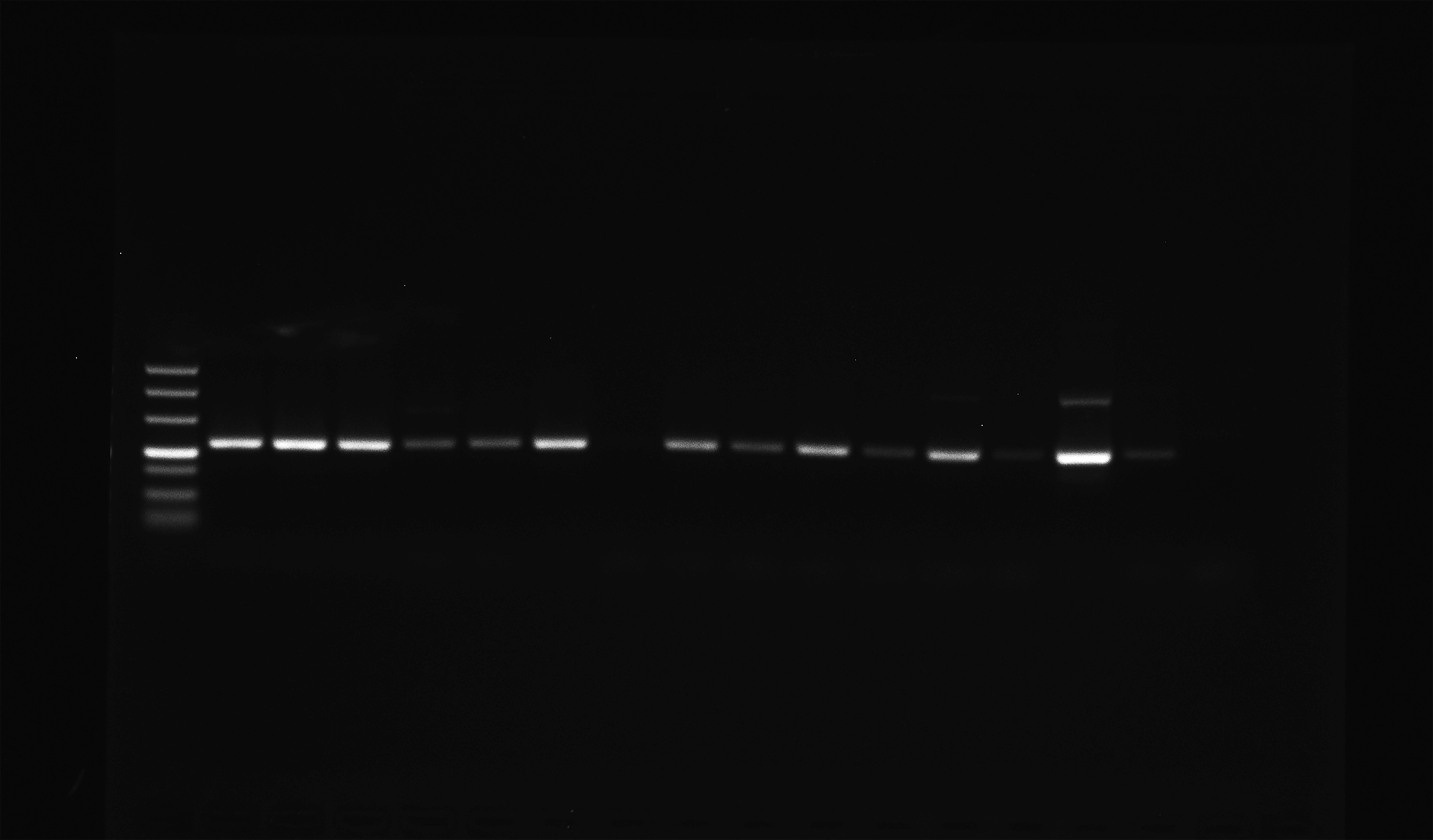


1. TRPM


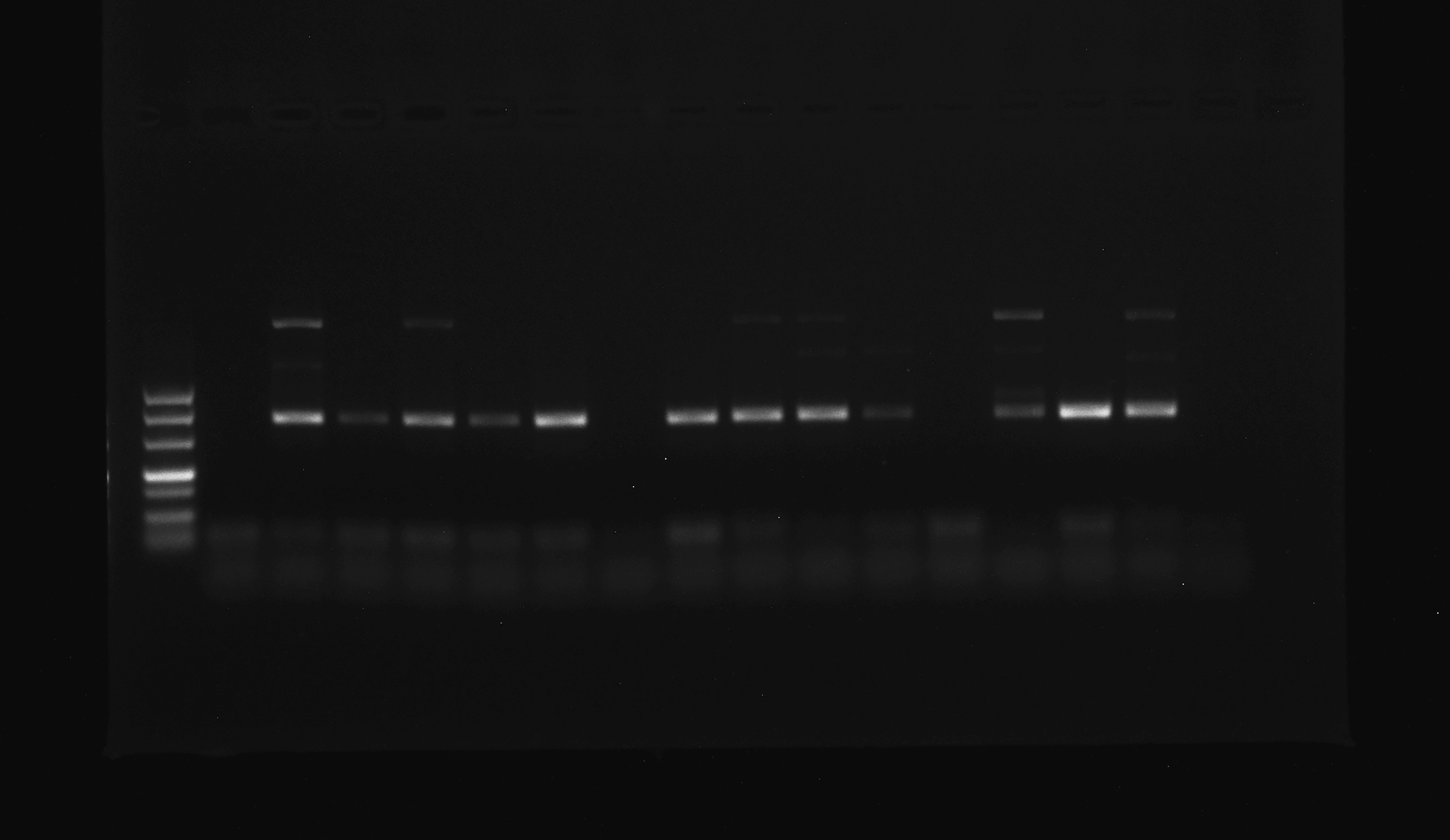


1. TRPML


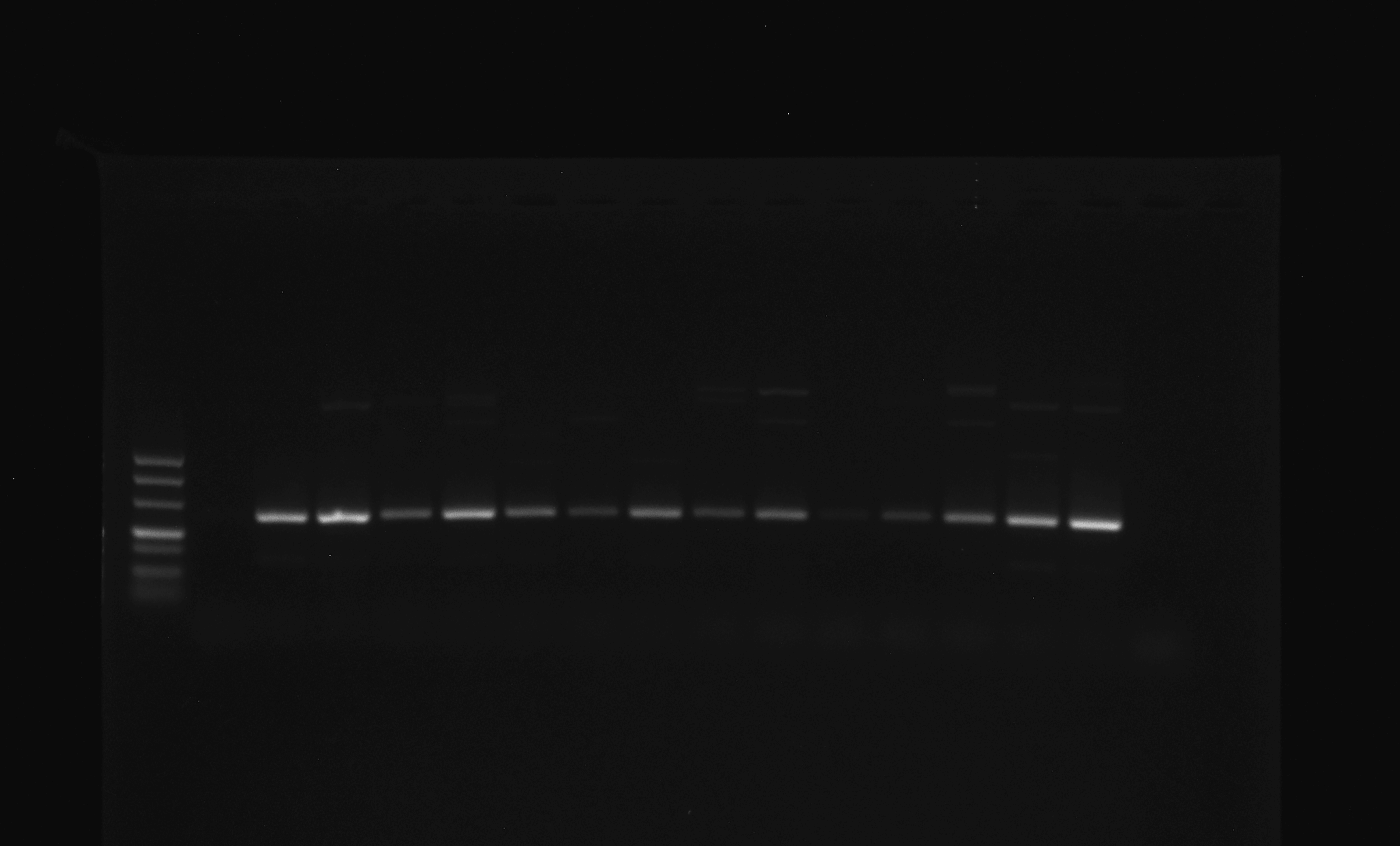


1. β-actin


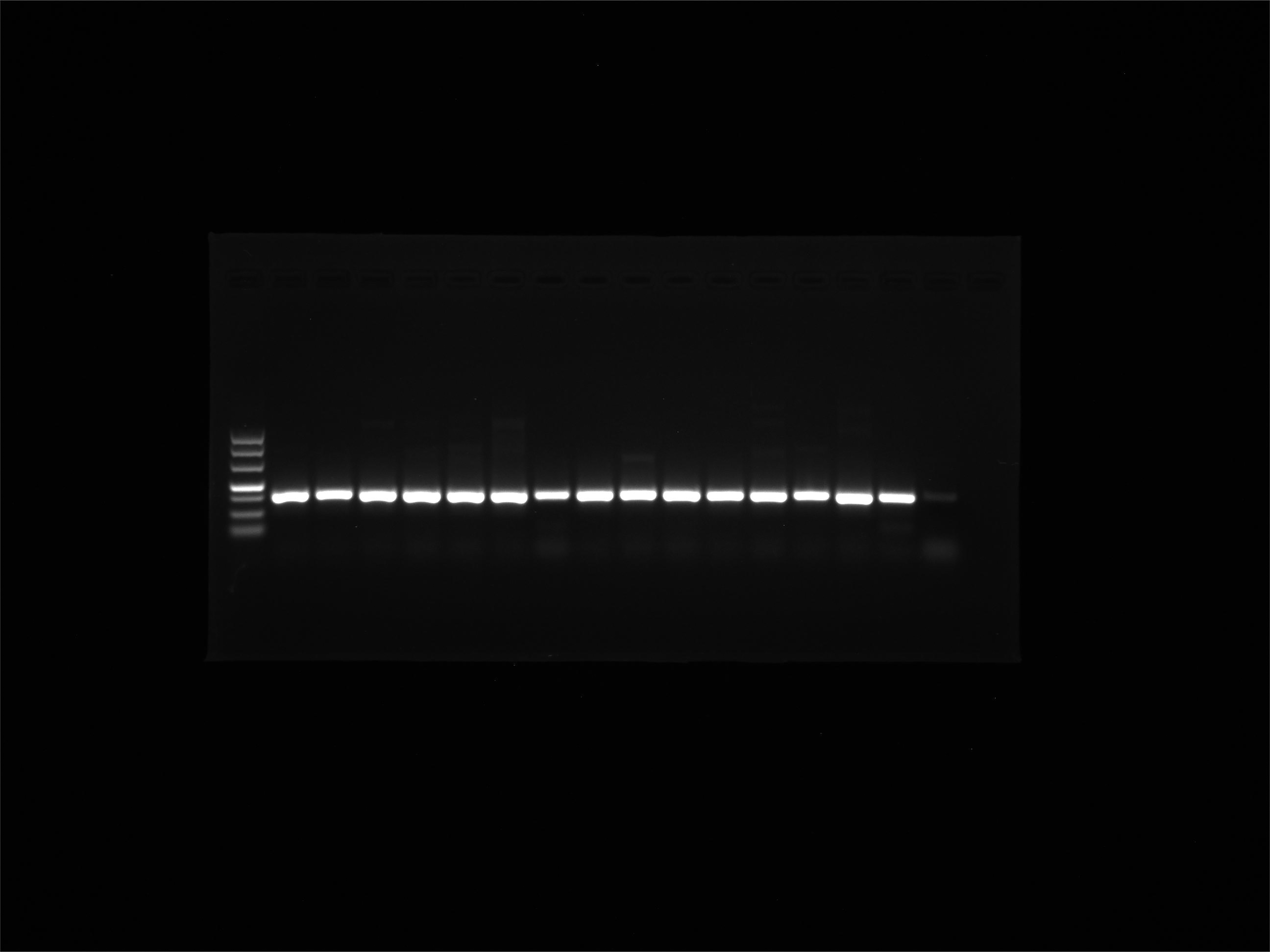

Supplement: Supplementary file 3 — Supplementary Material 3: Full-length gels which have been cropped in the main text [file 12864_2024_9973_MOESM3_ESM.docx]
